# Barriers to optimal care and strategies to promote safe and optimal management of sick young infants during the COVID-19 pandemic: A multi-country formative research study

**DOI:** 10.7189/jogh.12.05023

**Published:** 2022-09-03

**Authors:** PSBI Formative Research Study Group, Rasheda Khanam, Jennifer Applegate, Abdullah H Baqui, Arunangshu Dutta Roy, Salahuddin Ahmed, Main Uddin, Mohammod Shahidullah, Amha Mekasha, Abiy Seifu Estifanos, Damen Hailemariam, Dorka Woldesenbet, Nigussie Assefa, Lulu Muhe, Solomie Jebessa, Priyanka Adhikary, Nivedita Roy, Temsunaro Rongsen-Chandola, Nidhi Goyal, Vinod Sangal, Sarmila Mazumder, Nita Bhandari, Hina Mehrotra, Pramod Kumar Singh, Vinay Pratap Singh, Aarti Kumar, Vishwajeet Kumar, Yashwant Kumar Rao, Rupa Dalmiya Singh, Arun K Arya, Robinson D Wammanda, Laila Hassan, Ishakau Hassan, Emmanuel Ejembi Anyebe, Benazir Baloch, Imran Nisar, Nudrat Farheen, Sana Qaiser, Dania Mushtaq, Maryam Mansoor, Kiran Lalani, Fyezah Jehan, Rajiv Bahl, Karen Edmond, Shuchita Gupta, Sachiyo Yoshida, Yasir Bin Nisar, Shamim A Qazi

## Abstract

**Background:**

Essential health and nutrition services for pregnant women, newborns, and children, particularly in low- and middle-income countries (LMICs), are disrupted by the COVID-19 pandemic. This formative research was conducted at five LMICs to understand the pandemic’s impact on barriers to and mitigation for strategies of care-seeking and managing possible serious bacterial infection (PSBI) in young infants.

**Methods:**

We used a convergent parallel mixed-method design to explore the possible factors influencing PSBI management, barriers, and facilitators at three levels: 1) national and local policy, 2) the health systems, public and private facilities, and 3) community and caregivers. We ascertained trends in service provision and utilisation across pre-lockdown, lockdown, and post-lockdown periods by examining facility records and community health worker registers.

**Results:**

The pandemic aggravated pre-existing challenges in the identification of young infants with PSBI; care-seeking, referral, and treatment due to several factors at the policy level (limited staff and resource reallocation), health facility level (staff quarantine, sub-optimal treatment in facilities, limited duration of service availability, lack of clear guidelines on the management of sick young infants, and inadequate supplies of protective kits and essential medicines) and at the community level (travel restrictions, lack of transportation, and fear of contracting the infection in hospitals). Care-seeking shifted to faith healers, traditional and informal private sources, or home remedies. However, caregivers were willing to admit their sick young infants to the hospital if advised by doctors. A review of facility records showed low attendance (<50%) of sick young infants in the OPD/emergencies during lockdowns in Bangladesh, India (both sites) and Pakistan, but it gradually increased as lockdowns eased. Stakeholders suggested aspirational and pragmatic mitigation strategies.

**Conclusions:**

We obtained useful insights on health system preparedness during catastrophes and strategies to strengthen services and improve utilisation regarding PSBI management. The current pandemic provides an opportunity for implementing various mitigation strategies at the policy, health system, and community levels to improve preparedness.

The ongoing COVID-19 pandemic poses unprecedented challenges, particularly to low- and middle-income countries (LMICs), where health systems are unprepared for coping with such catastrophic situations. The quality of health care services suffers due to inadequately skilled health care personnel and a lack of equipment, medicines, and other supplies. The already over-burdened health systems in these countries have been further challenged during the pandemic, which disrupted essential health and nutrition services for the most vulnerable populations – pregnant women, newborns, and children [1,2].

Despite neonatal mortality decreasing over the last few decades, it still accounts for 46% of under-five deaths globally [3]. Preterm birth complications, intrapartum-related events, and infections are the most common causes of neonatal deaths worldwide [3]. However, in South Asia and sub-Saharan Africa, neonatal infections account for 35% of neonatal deaths [4]. The World Health Organization (WHO) recommends inpatient treatment for young infants <2 months of age with possible serious bacterial infection (PSBI) [5,6]. Studies have shown that these infants can be managed on an outpatient basis when referral to hospital is unfeasible [7-10] and have resulted in the development of new guideline [11,12]. Several studies have shown the feasibility and acceptability of WHO guideline on PSBI outpatient treatment in Africa and Asia [13-26]. Secondary analyses of clinical trials [7,8] have shown that infants with any sign of clinical severe infection had a higher mortality rate when hospitalised compared to outpatient treatment [27].

We conducted formative research to understand how the COVID-19 pandemic impacted the identification, care-seeking, and management of young infants with PSBI, specifically identifying barriers and corresponding mitigation strategies. The insights obtained from formative research would also be relevant for the planned PSBI trials [28,29].

## METHODS

### Study design and participants

The formative research used a mixed-methods design. We followed a convergent parallel design, where quantitative and qualitative data were collected concurrently to analyse the two components independently and interpret the results together [30]. We collected information iteratively at each level of the conceptual framework (Appendix S1 in the **Online Supplementary Document**) by interviewing respondents in each category to identify emerging themes and subthemes. New emerging areas were incorporated into the subsequent interviews, and the process was repeated until the point of saturation was reached.

### Procedures

The study was conducted in six settings: Bangladesh, Ethiopia, India (two sites: Himachal Pradesh (HP) and Uttar Pradesh (UP)), Nigeria, and Pakistan. The population consisted of individuals from low socioeconomic urban or rural backgrounds. The population size ranged from 300 000-5 000 000 (Appendix S2 in the **Online Supplementary Document**). Health care services in the study area were provided through public and private facilities and outpatient clinics, all of which had neonatal care units. Efforts were made to harmonise the study procedures by implementing a common standard protocol used from the outset through the conceptualisation stage, and proposal and study tools development, analysis, report writing, and manuscript preparation. All sites used a generic study protocol and standard study instruments. The site investigators analysed their data with inputs from all site investigators.

Trained and experienced social scientists and anthropologists collected qualitative data, while trained workers with health research experience gathered the quantitative data. Data collection varied across sites from December 2020 to March 2021. The quantitative data were collected retrospectively. The pandemic and subsequent lockdown period lasted for about three months at each site, from March/April to May/June 2020. All precautionary measures were taken against COVID-19 spread during data collection. The research assistants were checked for signs and symptoms of COVID-19 infection before their field visits.

For qualitative data, all sites used observations and in-depth interviews (IDIs), while focus group discussions (FDGs) were also conducted on all sites except two in India. Participants were selected from the entire geographical area served by the selected hospitals. Community health workers (CHWs) identified key informants (KIs). CHWs, staff nurses, medical officers, program managers, and most-visited private practitioners were interviewed. IDIs were conducted at various sites as follows: 12-49 with caregivers, 5-6 with KIs, 6-35 with CHWs, 5-14 with doctors, 6-12 with nurses, 4-10 with program managers and 3-7 with private practitioners. The IDIs continued until saturation was achieved and were all audio-recorded. Apart from HP and UP, all sites also conducted FGDs with caregivers and KIs. Data were obtained from 16-50 CHWs, and we surveyed 1-7 health facilities.

Quantitative data covering a preceding period varying from 9 to 12 months were collected retrospectively across sites from randomly selected CHWs and health facilities through observation, examination of health facilities records and CHWs’ registers. The key indicators by month were the number of sick young infants seeking care from outpatient departments (OPD), emergency services, or admissions and deliveries. The proportion of deliveries that occurred at home and the postnatal visits by CHWs during this time were ascertained. Closed-ended questionnaires were used.

### Analysis

Transcripts prepared from audio-recorded IDIs were reviewed to identify emerging themes and sub-themes, which were further extended for each target group. Relevant verbatim passages under each theme and sub-theme were extracted and translated into English. Framework analysis was used to examine the participants’ experiences and perspectives. The key themes were: identification of PSBI signs, care-seeking practices, treatment of sick young infants, barriers and enablers, the effect of the pandemic and lockdown on routine services, and mitigation strategies suggested by the respondents.

For the quantitative analysis, we explored barriers existing pre-COVID-19 and aggravated and new barriers during COVID-19. Simple means and proportions were estimated to examine the trends in service provision and utilisation of health care services. The quantitative and qualitative data were triangulated to understand the agreement across both.

### Role of the funding source

WHO received a grant from the Bill & Melinda Gates Foundation (#INV-001311). The funders had no role in the study design, data collection, analysis, interpretation, report writing or submission for publication.

### Patient and public involvement statement

Research question development was informed by the large burden of infection-related mortality among young infants worldwide. Patients were not advisers in this study, nor were they involved in the design, recruitment, or conduct of the study. Results of this study will be made publicly available through open-access publication where study participants may access them.

### Ethical considerations

Ethical approvals were obtained from the WHO Ethics Committee and the local ethics committees of all participating institutions. Each individual gave written informed consent for interviews and audio recordings.

## RESULTS

### Identification of PSBI signs

About two-thirds of caregivers listed high fever, inactivity, decreased movements, and inability to feed as symptoms requiring medical attention, but they did not report others, such as cold body and limbs (hypothermia), fast breathing, or noisy breathing. It was primarily evident among first-time mothers or those living in nuclear families. Elderly women/mothers with older children were more aware of signs requiring urgent attention (**Box 1** – Example 1).

Box 1 **Examples of verbatim quotes from qualitative data**

*Example 1 *Baby’s grandmother was telling me about the chest indrawing of the baby, and she instructed me to use home remedies for my child. We always listen to her.* (Caregiver, Ethiopia).*

*Example 2 *The fear that symptoms like cough, fever, breathing difficulties might be related to Corona, they tend to hide the information from others.* (CHW, UP, India).*

*Example 3 *People used to say, without showing the child, that the child is okay…were afraid of me and sometimes people even fight during visit…started calling me ‘Covid Wali’.* (CHW, UP, India).*

*Example 4 *My children were sick multiple times with fever, cough, and breathing difficulty during corona. However, my mother-in-law and sister-in-law suggested not to take the babies to the doctors as they will test the baby for corona, and they will isolate and separate my baby from me.* (Caregiver, Bangladesh).*

*Example 5 *We Pahadis (people who live in the mountainous regions) have enough strength to walk miles after miles (to reach the hospital), but how much pain and discomfort can a baby tolerate? It takes time to walk, and small babies do not have the power to suffer pain for that long, but we do not even have that much money to hire a vehicle to reach the hospital fast.* (Caregiver, HP, India).*

*Example 6 *Families do not bring the infants during the early phases of the illness. Since the cases come when they develop complications (or at a severe stage), they are not easy to manage at our health centre. So these families are forced to take the child to other higher facilities, or else the child will die.* (Health Provider, Ethiopia).*

*Example 7 *The entire pediatric outpatient department was turned into a COVID-19 unit due to lack of space and emergency because of which no babies could be treated.* (Medical Officer, Pakistan).*

*Example 8 *There was much fear due to the corona among the families, fear that corona testing itself would result in corona.* (Caregiver, UP, India).*

*Example 9 *We don't have any specific guidelines for managing infants in Kaduna State; although we have some national guidelines that can be domesticated, unfortunately, even domesticating the guidelines has always been the problem. …………However, we have guidelines for only a few conditions, for instance, treating very severe malaria, child transmission of HIV, cases of eclampsia and postpartum haemorrhage, which is distributed to all the hospitals.* (Health facility staff, Nigeria).*

*Example 10 *This time there is not much supply here. All the injections are being brought by patients only, and we are continuously sending indent for hospital supplies. We have been facing problems for more than one and a half months. One or two BP machines are out of order, IR thermometers we do not have…weighing scale we have…no instruments, one incubator, one side bed.* (Staff Nurse, UP; India).*

*Example 11 *Yeah, everyone was taking the child to a health facility, even during that time (Lockdown). I do not know anyone who kept his child at home due to COVID.* (Father, FGD; Adama, Ethiopia).*

During those times (lockdown), transportation had stopped, but I would visit the clinic by walking on foot and take a loan from my relatives to seek care.* (Caregiver, Bishoftu, Ethiopia).*

*Example 12 *What will happen now after COVID….no matter how the baby is…. this much mind has been made up that that work will be done after wearing gloves only, proper sterilization will be done…baby and family, both can have difficulty (infection).* (Health care Provider, UP; India).*

*Example 13 *…and with these people NURTW (National Union of Road Transport Workers, Nigeria), we try to procure some tricycles in collaboration with another project; we are able to procure this tricycle ambulance so we can establish it, but we had so many challenges ….in fact the voluntary collaboration with NURTW we can say is even better than the tricycle we distributed to all the political wards in the state, though the voluntary collaboration is mainly for pregnant women those that need attention.* (Official from Ministry of Health, Nigeria).*

*Example 14 *We tried not to stop the service by using digital technologies like creating telegram groups with regional focal groups and receiving technical support. We managed the protocols and documents to help the health post function. Moreover, we carried out a LEAP remote training platform for 3 or 4 days using their phones by SMS and Interactive Voice Response for HEWs on how to continue maternal and child health services provision during COVID.* (Policy Maker, Ethiopia).*

*Example 15 *In fact, the training was not restricted to health care providers. The findings indicated that the sensitization/orientation training was also given to the community, community and religious leaders.* (Health care Provider, Nigeria).*

CHW – community health worker, FGD – focus group discussion, HEW – health extension worker; HP – Himachal Pradesh, NURTW – National Union of Road Transport Workers; SMS – short message service; UP – Uttar Pradesh

Caregivers, KIs, and health care providers (HCPs) considered exposure to awareness generation activities as helpful. These included a mobile health service provided by the government of India [31], mother and child protection cards [32], community meetings, camps, and training sessions organised during the pre-COVID-19 period at different sites. Most caregivers across all sites depended on CHWs to identify and refer sick infants during the home-based postnatal care visits (**Table 1**). However, about one-fifth of CHWs could not list the danger signs requiring immediate referral. Most CHWs reported that identifying PSBI signs was challenging during the pandemic due to the disruption of home visits or assessing the infant without touching (**Box 1** – Example 2). CHWs also reported that caregivers were apprehensive about their visiting homes, suspecting them as COVID-19 carriers (**Box 1** – Example 3).

**Table 1:** Key pre-COVID-19 barriers, challenges, and facilitators, aggravated due to COVID-19 and new barriers and facilitors for identification of infants with possible serious bacterial infection (PSBI) by study site

Site	Barriers and challenges			Facilitators
	**Pre-COVID-19**	**Aggravated due to COVID-19**	**New**	
Bangladesh	• A few health care providers (HCPs) in public and private sectors noted that caregivers were not aware of signs of possible serious bacterial infection (PSBI). • Community health workers (CHWs) were unable to visit families during the lockdown.	-	-	• Mothers or mothers-in-law of caregivers were frequently cited as a support for identifying signs of illness. • All (12/12) caregivers reported that they were generally aware of the signs of PSBI with fever, cough, cold, and diarrhoea in young infants. Additionally, 8 (out of 12) caregivers could identify breathing and feeding difficulty. • CHWs and HCPs at primary health centres close to the community were cited as a source of referrals from the community to higher-level facilities.
Ethiopia	• Due to the high caseload volume in the pre-COVID-19 period, limited time was available for case identification. • The reluctance of parents to allow CHWs (health extension workers) to visit their infants even in the pre-COVID-19 period resulted in a delay in the identification of infants with PSBI.	• During COVID-19, the reluctance to allow CHWs was aggravated due to fear of getting exposed to a COVID-19 infection. • 5 CHWs (out of 8) reported that the routine services were affected due to the COVID-19 pandemic, and they were primarily engaged in awareness creation activities for the COVID-19 pandemic.	• Overlapping COVID-19 symptoms with the PSBI signs made it challenging for HCPs to distinguish between the two illnesses. • 5 CHWs (out of 8) reported that caregivers were reluctant to allow them to visit their infants. If they visited, they could not touch the infants for assessment. They were also asked to maintain a physical distance and not enter the home. • 7 CHWs (out of 8) said a lack of personal protective equipment (PPE) interfered with identifying sick young infants. • 3 HCPs in hospitals (out of 6) and 11 HCPs in health centres (out of 19) also mentioned the shortage of PPE. • Services were interrupted because staff either had a COVID-19 infection or was exposed to a COVID-19 patient. • The administrative staff was more focused on COVID-19 supplies than routine care. • One HCP (out of 25) mentioned a lack of motivation among HCPs. • Additionally, a family (out of 19) was hesitant to disclose their infant’s symptoms due to social stigma. As reported by CHWs, caregivers did not let them know about their sick infants, which led to a delay in identifying PSBI signs.	• Mothers were able to identify danger signs due to health education by CHWs and HCPs during antenatal care visits. • During the pre-COVID-19 period, 5-15 mothers (out of 20) were able to identify different PSBI signs at home. • 3 caregivers (out of 20) and two HCPs (out of 19) reported elderly family members as the primary source of identifying the signs of PSBI. • HCPs assessed infants cautiously because of the fear of COVID-19 infection. • Awareness was created in the community to identify symptoms related to the COVID-19 infection and other common illnesses. • Health centre staff conducted house to house visits to provide information on the COVID-19 infection. • A mobile application was used to increase awareness of the COVID-19 infection and the availability of essential services.
Himachal Pradesh, India	• 21 (out of 49) caregivers could identify only a few PSBI signs, but not all. Additionally, 9/49 caregivers and 3/5 HCPs said that family members and elderly people were the primary sources of identifying PSBI cases in most cases. • All caregivers reported that the CHWs, and HCPs, both in the public and private sector, never shared information on danger signs in young infants, even in the pre-COVID-19 period. • 4 (out of 49) caregivers and 9 (out of 18) key informants (KI) reported that the CHWs were unable to identify PSBI signs that needed medical advice, and the infant’s care was compromised due to delayed identification. • 6 (out of 31) CHWs said they were sometimes unwelcomed by the community members due to their frequent surveys and home visits. • CHWs complained about their workload. Many vacant positions, challenging geographical landscape, long-distance routes, snowfall, and bad road conditions disrupted their HBPNC visits. •	• 5 (out of 49) caregivers said that reliance on elderly members to identify PSBI signs increased during COVID-19 and lockdown, primarily due to fear of exposure and reduced visits by CHWs. • 13 (out of 49) caregivers shared their confusion over the runny nose, cough, sneezing, and fever, especially amidst the pandemic, as symptoms of severe illnesses or possible COVID-19 infection seemed similar. • 37 (out of 49) caregivers and KIs said that neither CHWs nor ANMs had shared information about danger signs, which they felt would be important during COVID-19. • 23 (out of 31) CHWs and all ANMs said that their workload was increased due to COVID-19, and they could not conduct the home-based postnatal care (HBPNC) and follow up visits. • 23 (out of 31) CHWs said that due to restricted availability of transportation during COVID-19, they needed to walk ‘miles after miles’ in the hilly terrain to conduct home visits which were often not feasible.	• 2 (out of 49) caregivers did not allow CHWs to come inside their homes due to fear of COVID-19 exposure. CHWs were considered carriers of COVID-19. • All (19/19) CHWs and 2 (out of 11) axillary nurse-midwives (ANMs) complained about inadequate supply of masks and that no PPE was given to them; they felt unprotected, interaction with caregiver and examination of baby was therefore restricted or compromised. • 3 (out of 49) caregivers said they received no advice from HCPs after delivery as there was a rush to discharge mothers due to the COVID-19 situation. In the pre-COVID-19 times, they were informed about conditions when they needed to seek care from the hospital at the time of discharge post-delivery. • CHWs felt insulted for being discriminated against by the families and their rude behaviour. • CHWs were worried about their safety, their families, and those they visited. They were scared of being attacked by wild animals while travelling through deserted forest areas during the lockdown. • CHWs were demotivated, and no incentives were paid for their COVID-19 duties. • No information was given on disposing of used masks and gloves. They threw the used items in the open in the backyard of the facility.	• Mother and child protection card and *Kilkari*, which is an audio-based maternal and child health messages service, were considered helpful for the identification of symptoms. • Elderly family members and experiences of child-rearing also helped. • The consensus was that burden of illnesses was reduced during COVID-19 due to improved sanitation and hygiene practices, quality time spent with the infants and children at home, better childcare, social distancing and reduced interaction, unpolluted environment, increased awareness of zinc in diarrhoea, parents, more vigilant due to overlapping symptoms of COVID-19 and PSBI. • Regular meetings and camps were conducted for awareness. • Faith and trust in CHWs and the availability of mobile phones with the families were positive aspects. • Program managers did not perceive any challenges in identification. • Families called immediately or went to the hospital for infant illnesses.
Uttar Pradesh, India	• 14 (out of 19) caregivers were unable to assess danger signs at the early stages of illness. • First-time mothers lack abilities to assess their infants and identify symptoms. • No HBPNC training of urban CHWs took place, as stated by all (10/10) urban CHWs. • 23 (out of 25) CHWs reported a lack of necessary equipment such as a thermometer, stopwatch for counting respiratory rate, and lack of training to calibrate equipment as barriers to identifying danger signs. • In-migrants who did not have maternal and child health cards did not get registered and missed out on services. • Despite HBPNC training, rural CHWs lacked skills and were not confident in identifying PSBI signs. • Change in residential address of beneficiaries creates hurdles in rapport and trust establishment.	• Increased concerns of caregivers were observed. 13 (out of 19) caregivers reported fear of COVID-19. They did not let anybody touch their infants, whether they were family members or outsiders. CHWs, therefore, failed to assess the health of the infants. • Additionally, CHWs were asked to do contactless visits; they were instructed not to touch babies. Therefore, all CHWs (25/25) said that they did not even enter homes or touch the doors. They would only ask the parents from a safe distance about the infants’ well-being and, therefore, could not assess the temperature and weight of the infant or support mothers. Parents did not take out the infants and show them to the CHWs.	• CHWs were provided only one mask and one bottle of sanitiser for their fieldwork, and as part of their COVID-19 duties, they were regularly exposed to COVID-19 positive patients. • Families hesitated to share information about their infant’s illness in the early stages due to a fear that symptoms like cough, fever, and breathing difficulties may correlate with COVID-19. There was a widespread fear of institutional quarantine and social isolation. • Post-birth traditional rituals ceased during the lockdown, which missed opportunities to get guidance from elder relatives.	• Mothers and elderly family members can typically identify if anything is wrong with their baby, although the main challenge is early identification. Symptoms mentioned as alerts included fever, refusal to feed, excessive crying, breathing difficulties, grunting, differences in the crying pattern, change in colour/ jaundice, and frequent passing of stool. • Due to COVID-19, alertness in recognising signs increased, and other illnesses presenting with similar symptoms were also getting identified. • Local untrained health service providers and faith healers are available close to home. • CHWs are contacted over the phone by caregivers. • Awareness through media advertisements on COVID-19 was beneficial. • Infections due to contact at home were reduced, and handwashing and use of sanitiser increased.
Nigeria	• At the secondary health facilities, health care providers considered the lack of guidelines from the Ministry of health as a major factor contributing to the identification of symptoms of PSBI. • Parents expect the house to house visits by CHWs for PSBI identification and management, which is not feasible.	-	• The emphasis placed on patient screening for COVID-19 had affected the identification of young infants with signs of PSBI. • Disharmony was created among health care providers regarding classification as frontline/non-frontline for payment of incentive (New COVID-19 hazard allowance) as some HCPs in the facilities were left out.	• In the pre-COVID-19 period, 3 (out of 15) caregivers were able to identify signs of PSBI; 2 (out of 15) caregivers said that they relied on experienced grandmothers to identify signs of PSBI in their newborns. Only 1 (out of 15) caregivers reported relying on the CHWs to identify PSBI signs. • Coverage of community testing was increased as a deliberate policy to allay the fear of COVID-19. With many people being tested, the fear that every sick person will likely have COVID-19 will be reduced. • Volunteers were engaged in reducing the impact of staff shortages in the community.
Pakistan	• Only 8 (out of 28) caregivers were able to identify danger signs of PSBI. 6 (out of 14) HCPs could identify PSBI signs. • 3 (out of 6) CHWs reported that some parents and family members resisted seeking help from CHWs to identify PSBI as they thought the illness was too severe for them to manage. It was beyond their capability. • Visits by CHWs were rare in some pockets of the population.	• Some fathers denied the existence of COVID-19. • There was confusion due to a lack of clarity between COVID-19 and PSBI signs, which aggravated the delay in identification. 20 (out of 28) caregivers were confused between PSBI signs and COVID-19 signs, reducing the number of caregivers identifying PSBI signs.	• CHWs could not hold regular health sessions due to prohibitions on social gatherings, and their everyday work was disrupted. • Caregivers prevented the CHWs from entering their homes for postnatal visits and wanted doctors to visit homes instead due to fear that the CHWs were potential carriers of COVID-19, as reported by 5 (out of 6) CHWs.	• Caregivers depended on the elderly women of the household, neighbours and CHWs, who could identify danger signs. There was no hesitation from CHWs to visit homes. • CHWs were trained to identify danger signs; they had an internal monitoring system, and they had to record and report the status of babies to their supervisors.

ANMs – auxillary nurse-midwives, CHW – community health worker, HCP – health care provider, HBPNC – home-based postnatal care, KI – key informants, PPE – personal protective equipment, PSBI – possible serious bacterial infection

Most HCPs in Nigeria and a few in Bangladesh, Ethiopia, and HP, India, said a shorter post-delivery stay at the health facility reduced the opportunity of orientating mothers about danger signs. A large proportion in southeast Asia and a few Nigerian caregivers reported that families concealed the information about their infants’ illness from CHWs due to fear of COVID-19-related stigma and isolation in the hospital. Whereas one-third of caregivers from India and Nigeria reported seeking care immediately because COVID-19 and PSBI symptoms overlapped. About three-fourths of HCPs and program managers in Indian sites and a few in Africa reported a reduction in other common illnesses. Facilitating factors were improved hygiene practices, restricted social gatherings and movement outside the home, absence of birth rituals, quality time spent with children, close watch on infants, low pollution levels, and media promotion to prevent other common illnesses (**Table 1**).

### Care-seeking practices

Most caregivers at the southeast Asian sites and around half at the African sites tried home remedies in the early stages of illness, seeking outside care only when the infant was unresponsive (**Table 2**). Four-fifths of mothers reported their husbands and parents-in-law not allowing care-seeking from hospitals. Women with multiple children or those living in nuclear families found it challenging to travel alone to the health facility or admit their infants. In UP, India, three-fourths sought care from informal (untrained) private providers. Care-seeking outside the home was affected during the lockdown. One-fifth of caregivers at all sites (apart from UP, India) preferred the private sector and local pharmacies, as they perceived them safer than government hospitals in this pandemic. Furthermore, government facilities were either closed or had restricted opening times and services available in OPDs. Caregivers avoided government facilities due to mandatory COVID-19 testing, which resulted in delays in receiving treatment. Most also said they could not afford private care (**Box 1** – Example 4).

**Table 2:** Key pre-COVID-19 barriers and challenges aggravated due to COVID-19 and new barriers and facilitators for care-seeking and referral during by study site

Site	Barriers and challenges			Facilitators
	**Pre-COVID-19**	**Aggravated due to COVID-19**	**New**	
Bangladesh	• 10 (out of 12) caregivers reported that long-distance was associated with reduced access to health facilities, both in public and private hospitals. • Women have limited autonomy in decision-making about care-seeking. 9 (out of 12) caregivers reported that the father of the child and grandmothers made decisions. • All caregivers (12/12) opted for wait and see approach in the initial stage of PSBI after identification, which resulted in administering home remedies or visiting a traditional healer or homoeopath at the first signs of illness in their babies.	• Lack of transportation was aggravated, which reduced the care-seeking from the hospitals. • Caregivers resorted to home remedies and from the nearest medical pharmacies if sickness was aggravated. • Though the care-seeking was low during the COVID-19, doctors in public and private sectors reported that referrals from lower-level facilities were high due to the severity of illness and COVID-19 infection, thus leading to delayed care-seeking.	• 10 (out of 12) caregivers expressed their apprehensions around visiting hospitals due to exposure to COVID-19 infection and fear of COVID-19 test that would lead to extended quarantine and stigma if positive. • 6 (out of 20) health care providers (HCPs) in public and private sectors reported that families did not seek care outside the home for sick young infants during the pandemic, primarily due to fear of exposure to COVID-19 and financial crisis due to unemployment.	• High coverage of mobile phone usage allowed community health workers (CHWs) to follow up with families in the community.
Ethiopia	• CHWs reported that mothers preferred home remedies in the early stage of PSBI. 2 (out of 8) CHWs and 1 (out of 20) caregiver reported using home remedies at the initial stages of PSBI.	• 1 (out of 20) caregiver reported use of home remedies increased during the COVID-19 pandemic because of fear of getting an infection at a health facility. • Admission rooms for infants were overcrowded, no treatment (medicine) except oral amoxicillin was available at the health post, and long waiting times were some of the factors affecting care-seeking even before COVID-19. • Mothers refused referral due to economic problems and other children to take care of at home. • The number of accompanying attendants during referral was reduced during the COVID-19 pandemic.	• Shortage of transportation affected caregivers’ and HCPs access to health facilities and transportation of medicines and supplies. 13 (out of 20) caregivers and 3 (out of 19) HCPs reported transportation constraints as a barrier to care-seeking. • Caregivers were afraid of visiting health facilities due to the COVID-19 infection. 8 (out of 19) HCPs reported that the caseload decreased due to fear of the COVID-19 infection. • 4 (out of 20) caregivers reported that due to fear of COVID-19 infection, they did not take sick infants to a public hospital. • 5 (out of 19) HCPs reported referral rates were low during lockdown because of low outpatient department (OPD) and inpatient department (IPD) footfall. • 11 (out of 19) HCPs reported the shortage of medical equipment during the COVID-19 pandemic in the facilities, possibly leading to reduced care-seeking.	• Good referral linkage between health centres and hospitals, including getting weekly feedback from the hospital. • Parents did not prefer traditional medicines for infants. • Parents were seeking care based on the information they received from health care providers at health centres, CHWs, and the health development army in the community.
Himachal Pradesh, India	• Almost all caregivers preferred higher-level health facilities due to the availability of all necessary provisions and easier direct referral from there if needed, although it was not easy to access. • Challenges in care-seeking existed even before COVID-19, inappropriate location of the facilities, inadequate provision of specialised care for sick infants, overcrowded hospitals, long waiting time, lack of HCPs, nurses, non-availability of medicines for young infants at primary health centres, community health centres and Civil hospitals, limited hours of service available at the lower-level facilities, long-distance, lack of transportation and inadequate ambulance services, hilly terrain, inaccessible roads. All these factors, particularly hilly terrain and the inability of ambulances to travel inside the village, led to delays in seeking care. 29 (out of 49) caregivers, 15 (out of 18) KIs reported these. • 19 (out of 49) caregivers complained about inadequate ambulance services and the poor condition of the available ambulances. • 31 (out of 49) caregivers relied on home remedies and waited for 1-2 days for the infant to recover. They only sought care from facilities when their health condition worsened. • 9 (out of 49) caregivers went to faith healers due to trust in them. • 8 (out of 49) caregivers reported that they were compelled to go to a private hospital because of a lack of appropriate provision for investigations such as ultrasound, blood tests and radiographs in public health care facilities. • 10 (out of 49) caregivers preferred private hospitals over public hospitals as they had greater faith in the private doctors. • 9 (out of 49) caregivers reported that due to overcrowding in the tertiary hospitals, they avoid travelling a long distance to go to these hospitals and visit the nearest local private clinic, which is more convenient.	• Caregivers were apprehensive of visiting hospitals or using the ambulance for fear of contracting the disease. • Besides, the doctors were reluctant to touch the infants and examine them appropriately. • In many families, the father or other male members did not allow infants to be taken to hospitals. They bought medicines from the local pharmacy by narrating the symptoms of the current illness. • Public transportation became scarce, and private transport became more expensive than usual.	• Financial difficulties increased due to loss of employment. Therefore, home remedies and seeking care from faith healers during the pandemic increased, as reported by 5 (out of 21) KIs and 35 (out of 49) caregivers. • 32 (out of 49) caregivers were satisfied with home remedies that worked better than other medicines. • A shift of care-seeking to the private sector, alternate systems of medicine, informal sector and faith healers were observed, as these were perceived to be safer than the government facilities. • 7 (out of 31) CHWs said that their routine services for the care of infants were compromised as they were assigned COVID-19 related duties.	• The community has strong faith in the government health system and willingly admits their children, irrespective of all the barriers if advised. • Village administrative heads and neighbours are helpful and arrange for transport in emergencies. • Toll-free phone service is available, which could be used to inform if anyone has a fever. • In case of an emergency, people could get a pass made quickly and go to the hospital during the lockdown. • Even during the lockdown, medicine shops were open 24 h. • During COVID-19, some parents became very cautious, and they went to the doctor even if the child showed some signs of illness.
Uttar Pradesh, India	• A birthing facility is typically the preferred choice for care-seeking for the baby. 12 (out of 25) caregivers reported that ambulance services were available only for severe cases and government referral facilities are usually further away than private facilities. These factors affect care-seeking from government hospitals. • 17 (out of 25) caregivers shared that they sought care initially from faith healers but were inhibited from sharing this information with HCPs, and this delays care-seeking and makes it difficult without prior records or case history. • All HCPs in the Community health center said no provision to admit newborns and infants at the Community health center level. • 2 (out of 3) HCPs shared that no appropriate linkage exists across the different government level health facilities, ie, primary, secondary and tertiary. • All HCPs said that sick newborns were not admitted or screened at the Community health center at the emergency, leaving only referral as an option. • 8 (out of 25) caregivers said that it was time-consuming to seek care from secondary government facilities due to overcrowding and the harsh behaviour of facility staff. This discouraged caregivers from accessing secondary health facilities unless necessary. • Care-seeking from multiple informal providers delayed care-seeking from appropriate sources and led to a lack of appropriate treatment of sick infants. 5 (out of 25) caregivers sought care from informal providers. • Many untrained and uncertified informal HCPs set up clinics in the local markets, homes or run their mobile clinics. Educated patients avoided going there as they provided irrational medicines and antibiotics. • 10 (out of 25) caregivers reported that families prefer their contacts and network in seeking care for children. The key reason was CHWs’ lack of familiarity with referral facilities. Additionally, ill behaviour by hospital staff prevented care-seeking.	• 15 (out of 25) caregivers said that families deliberately delayed seeking care from formal providers due to COVID-19 fear till they could manage with home-based remedies or from the nearest local informal providers.	• Several public sector hospitals were converted into COVID-19 facilities. Designated COVID-19 hospitals could not serve non-COVID-19 patients. Partial COVID-19 hospitals were not fully equipped to handle tertiary-level non-COVID-19 cases. • Information on the availability of services or beds during COVID-19 in public health facilities was inadequate. • Care provision in government hospitals was delayed due to the imposition of COVID-19 testing as a pre-requisite for admission and outpatient care. No policy for the regulation of untrained health service providers was in place. • All the CHWs said that responsibilities increased. In addition to their routine work, CHWs were engaged in the COVID-19-related work, including surveys, institutional isolation of patients in their respective areas, and monitoring and supporting the migrants who were quarantined for 14 days. All these interfered with the CHW availability and access and hence care-seeking. • 11 (out of 25) caregivers prefered local private HCPs over public health facilities, for prolonged symptoms such as cold/cough, fever, stomach ache, reduced feeding, chest indrawing, and jaundice, due to social stigma associated with COVID-19 in public hospitals.	• Private HCPs capitalised on health system challenges to provide more responsive services, attract more clients, and plug the gap in service delivery in the health system. • The private HCPs adjusted their clinic timings according to patients’ convenience, continued to provide all regular services, improved their support to patients and sometimes also provided home services.
Nigeria	• All HCPs reported a lack of provision for newborn admission, which affected care-seeking. • The bed spaces for inpatient services and paediatric wards were limited for young infants. Therefore, families did not seek care from health facilities. • All facility In-charges and CHWs reported a poor two-way referral system, described as non-functional and uncoordinated, and a limited number of referral centres. Transport arrangements were poor. • Additionally, most caregivers reported wrong perceptions, false beliefs, and fear about poor prognosis at referral level tertiary facilities. • Many mothers were uncomfortable being attended to by male health care workers, thereby restricting their access to seek care for their infants. • Seeking spouse approval of hospital visits was another factor influencing care-seeking behaviour. • The financial crisis and poor economic conditions led to reduced care-seeking from hospitals	• HCPs from 2 (out of 4) government health facilities reported a further reduction of the already limited bed spaces in the paediatric department, as the beds were shifted to the COVID-19 department to meet up with the COVID-19 guidelines concerning hospital admissions.	• Further reduction of care-seeking due to shorter duration of consultation at hospitals was reported, which the nurses and doctors expressed at one of the secondary health facilities. • Patient screening for COVID-19 resulted in fear of social stigma among caregivers, reducing care-seeking. • All 15 caregivers reported fear of hospital visits during COVID-19. • The shift in care-seeking from facilities to home remedies and faith healers happened during COVID-19.	• Mandatory screening and adherence to COVID-19 preventive measures of patients and visitors at the hospital entrance created confidence among families. • The 255-ward services policy was designed as the first level referral for primary health care facilities, thus reducing the need for referral to very far off places. There are 255 wards in Kaduna state. The policy was to have at least one functional Comprehensive Health Center that should receive referrals from the immediate primary health centers within the wards. So, one of the primary health centers (or the only primary health center) in each ward was upgraded to the level of Comprehensive health centre as the first referral level for the ward.
Pakistan	• 2 (out of 7) policymakers said crowding and lack of trained staff in public health facilities led to a shift in care-seeking to private facilities or resorting to home remedies. • 9 (out of 28) caregivers reported that they sought care from the local private clinics because of the low quality of care in the public facilities at secondary or tertiary levels. However, 15 (out of 28) caregivers reported seeking care from government clinics. • 8 (out of 14) HCPs reported that most sick infants were referred to another hospital due to inadequate care provision.	• 3 (out of 28) caregivers said private hospitals became inaccessible due to financial difficulties and increased prices.	• 2 (out of 6) HCPs said there was reluctance amongst the community members in getting treatment. People were also avoiding CHWs in addition to avoiding hospitals. • Patient screening for COVID-19 resulted in fear of social stigma among caregivers, reducing care-seeking. The shift in care-seeking from facilities to home remedies and faith healers during COVID-19 was mentioned by 11 (out of 28) caregivers	• The mother’s autonomy in taking decisions regarding care-seeking was not a major concern, and all mothers reported that no one in the household ignored the sick baby. • Caregivers approached CHWs during the lockdown period to get medicines. Even when CHWs could not conduct household visits, caregivers would bring their babies to the health centre in their area themselves. • Most caregivers reported letting CHWs enter their house since they were familiar, and CHWs continued doing household visits. • The majority reported government hospitals being their primary source of seeking care. • HCPs were also provided adequate PPE.

ANMs – auxillary nurse-midwives, CHW – community health worker, HCP – health care provider, IPD – inpatient department, KI – key informants, OPD – outpatient department, PSBI – possible serious bacterial infection

Travel restrictions, non-availability of transport, increased police surveillance and interrogation, a significant financial strain for families, especially those who had lost jobs, reduced quality of care, and unpredictable staff availability at government facilities were also barriers to care-seeking (**Table 2**). Some of these existed before the pandemic (**Box 1** – Example 5).

### Treatment, adherence and follow-up of sick young infants

Two-thirds of the facility staff across all sites stated that inadequate human resources and staff shortage due to COVID-19 duties or quarantined staff, insufficient supplies of oxygen and medicines for young infants, and unhygienic conditions in the facilities led to reduced utilisation of facilities and sub-optimal management of young infants, in both OPD and inpatient facilities (**Table 3**).

**Table 3:** Key pre-COVID-19, aggravated due to COVID-19 and new barriers and facilitators for inpatient and outpatient treatment, compliance and follow-up care by study site

Site	Barriers and challenges			Facilitators
	**Pre-COVID-19**	**Aggravated due to COVID-19**	**New**	
Bangladesh	• 4 (out of 6) policymakers and 10 (out of 20) health care providers (HCPs) in public and private hospitals reported that the infants were given paracetamol and antibiotics from unregulated pharmacies before coming to hospitals. This interfered with receiving appropriate treatment from suitable sources. • 8 (out of 12) caregivers reported long waiting times, inadequate supply of medicines, uncleanliness, overcrowding and lack of attention by the health care providers as common challenges at the outpatient departments (OPDs). • Complying with treatment and following referrals was challenging for mothers from low-economic backgrounds. • Caregivers reported that the community health workers (CHWs) came only twice at home to visit the infants for postanal care visits.	• 10 (out of 12) caregivers had trouble reaching the hospital OPDs due to restricted transportation that was exacerbated by COVID-19. Caregivers resorted to seeking care from local doctors. • 8 (out of 14) HCPs working in public sector sub-district/tertiary hospitals reported a limited number of incubators, lack of a bed, and investigative facilities to provide appropriate care to young infants.	• 3 (out of 12) caregivers who sought inpatient care for their infants reported that very few doctors were available and there were medicine stock-outs. • 11 (out of 14) HCPs in the public sector and 13 (out of 16) CHWs reported that routine services such as vaccination, antenatal care and postpartum care were affected during the lockdown period. • 8 (out of 12) caregivers reported insufficient supplies of oxygen and medicines in the hospital. They also mentioned very brief interaction with the doctors, who did not even care to touch the infants and examine them closely. • All (16/16) CHWs reported that community clinics were closed during the lockdown. CHWs came only once for a home visit during COVID-19. • Both policymakers and providers noted delays in receiving PPE.	• Families reported taking advantage of microcredit loans from the local non-government organisations or using savings to pay for infant care.
Ethiopia	• 2 (out of 6) hospital HCPs reported a lack of beds and space for the paediatric ward, and low quality of care as challenges in the provision of inpatient care. • Shortage of medical equipment and supplies was a barrier even before COVID-19. 11 (out of all 25) HCPs reported a shortage of oxygen, medicine, investigation modalities, manual sucker and nasal prong, even before COVID-19.	• 1 (out of 6) hospital HCPs reported a shortage of beds and space increased due to the repurposing of space for COVID-19 care and the repurposing of a few hospitals to COVID-19 centres. • 3 (out of 6) hospital HCPs reported that breastfed infants who did not need oxygen/supportive care were discharged within 48 h after delivery due to COVID-19 fear. • 5 (out of 6) hospital HCPs reported that a few existing staff were shifted full-time to the COVID-19 centres, while the remaining were engaged for COVID-19 care part-time, which led to a shortage of staff availability to non-COVID-19 patients. • 2 (out of 20) caregivers reported that the OPD in a few hospitals for under-5-y-old children was closed down and used for emergency and delivery care during the COVID-19 pandemic. • All caregivers (n = 20) reported that CHWs did not make a home visit for postnatal care. However, only 2 (out of 20) caregivers reported that due to the COVID-19 fear, CHWs came only once for home visits. • 5 (out of 20) caregivers reported that they were afraid of the COVID-19 exposure and hence refused to comply with referral advice to tertiary care facilities. • Follow up care was inadequate even before the COVID-19 pandemic. One hospital HCP (out of 6) reported that more young infants from other regions (out of their catchment area) presented to their hospital than in their catchment area.	• Complying with treatment and following referrals was challenging for mothers from low-economic backgrounds even before the COVID-19 pandemic. 2 (out of 19) facility HCPs and 2 (out of 20) caregivers reported financial constraints as one of the barriers. • The caregivers’ understanding was poor for follow-up advice mainly due to the non-corporation of HCPs.	• Mothers preferred health facilities for sick young infants. Parents accepted treatment and advice given by HCPs. High compliance with the referral and follow up appointment by the HCPs. • The linkage between local health centres and health posts was essential for the follow-up visits. • CHWs (Health extension workers) called parents of sick young infants referred to health centers for further management to check whether the referral advice was followed and treatment was received from health centers. • Limited visitors/ attendants allowed in facilities. • Water pipes and other equipment at health facilities were maintained well. • Improved cleanliness of the delivery room, good hygiene and sanitation practices. • Bed-sharing for infants was stopped.
Himachal Pradesh, India	• Sub-optimal care at lower-level government health facilities; provisions such as investigations, blood tests, radiographs, ultrasounds, advanced “machines” (ventilators, warmers), essential medicines and supplies are not available. A referral is difficult. 17 (out of 49) caregivers said there was inadequate provision for advanced care of sick infants in the Civil Hospital (secondary), such as warmers and ventilators. 4 (out of 6) staff nurses said that because of the lack of ventilators or monitors in the paediatric ward in the hospital they were unable to provide care to the infants. • Almost all the private HCPs said it was a big challenge to sustain qualified doctors in remote areas. It was not easy to sustain qualified doctors in the district. • 1 (out of the 3) HCPs said that low pay scales, contractual nature of jobs, lack of good schools for their children, appropriate health care facilities in case of emergency, cut off from social life, lack of any recreational activities, poor working conditions etc., were demotivating factors. All these indirectly affect patient care, treatment and counselling by the doctors for compliance with treatment. • The private HCPs did not manage sepsis in young infants. • Lack of clear guidelines on newborn care and management of PSBI cases, essential newborn care, particularly for low birth weight or preterm babies, breastfeeding, the do’s and don’ts, the protocol to be followed for infants with symptoms, etc. cited as a challenge. • 9 (out of 49) caregivers said that few primary health centers remained closed and non-functional even before COVID-19. • For outpatient care, 20 (out of 49) caregivers said that medicine supplies at the sub-centres and primary health centers were inadequate, and most medicines were not available. • 28 (out of 49) caregivers said the waiting time at the study hospitals was too long. • 39 (out of 49) respondents said that there was a lack of staff across all levels of health facilities, primary, secondary and tertiary. There are 2 doctors in the secondary level hospitals for attending to both OPD and inpatient with only a few staff nurses. • 19 (out of 49) caregivers said that there was a lack of pharmacists in the PHCs for outpatient services and, primary health centers were not open 24/7, no staff was available at night. • Regarding compliance, 1 (out of 49) caregivers said that treatment compliance for young infants was affected due to long-distance travel from residence to a tertiary or referral speciality hospital. • Regarding follow up care, 5 (out of 31) CHWs and axillary nurse-midwives complained about their workload. • 11 (out of 31) CHWs deplored keeping several positions vacant, which they felt was a major impediment to follow up care. • 3 (out of 31) CHWs said that challenging geographical landscape, long-distance routes, snowfall, and bad road conditions disrupted postnatal home visits.	• Primary health centers, and community health centers pediatric ward were closed during the lockdown. 22 (out of 49) caregivers said that they could not avail treatment in the hospital because the paediatric ward was closed during the lockdown. All emergency referrals were getting treated in the emergency ward. • Caregivers were unsure of visiting hospitals as they did not have clear information on which hospitals were either entirely or partially converted to COVID-19 care centres. • 29 (out of the 49) caregivers said that medicine supplies at the sub-centres and primary health centers were inadequate. • 35 (out of 49) caregivers said that doctors were available in very few primary health centers during lockdown because they were busy doing COVID-19 duties. • 16 (out of 49) caregivers said the OPD load rapidly reduced after the news of COVID-19 infection amongst a few OPD staff. Inpatient admissions were discouraged by the facility staff, including doctors. • 19 (out of 49) caregivers said that the mothers and babies were discharged soon after delivery during the lockdown due to the COVID-19 fear. • 3 (out of 19) caregivers said that preterm infants delivered through cesarean section cases were also discharged early. • During the inpatient admission, neither the staff nurses nor the doctors instructed the mothers appropriately on PSBI signs and actions to be taken. • 11 (out of 49) caregivers said that the hospital staff were behaving rudely toward them. Due to this, the quality of care was compromised. • 3 (out of 49) caregivers experienced extremely rude behaviour by the staff nurses and the doctors during the lockdown. • 9 (out of 49) caregivers refused inpatient treatment at the community health centers due to poor hygienic conditions, which was not the case before lockdown.	• Lack of adequate human resources in facilities was a major challenge, and staff had to multitask with long duty hours. • Hospital staff faced discrimination in their society and neighbourhood for working in hospitals. 1 (out of 6) staff nurse reported discrimination. She and her child were singled out in the neighbourhood as she worked in a hospital during the lockdown and the pandemic. • HCPs and CHWs expressed discomfort working with a PPE in heat and humid weather conditions. • 5 (out of 6) staff nurses revealed their apprehensions about reporting to duty in the facilities due to the fear of contracting COVID-19 from workers, including sweeper and support staff. • 16 (out of 49) caregivers revealed sudden employment and financial challenges disrupted compliance with treatment and follow up care. • Regarding the provision of follow up care 23 (out of 31) CHWs and axillary nurse-midwives said their workload was increased due to COVID-19, and they could not conduct the postal home follow up visits. • 23 (out of 31) CHWs said that due to restricted availability of transportation during COVID-19, they needed to walk miles after miles in the hilly terrain to conduct home visits which were often not feasible. • 12 (out of 49) caregivers did not allow CHWs to come inside their homes due to fear of COVID-19 exposure. The CHWs were considered carriers of COVID-19.	• Emergency services in the hospitals were functional round the clock. • Compliance with treatment, referral, and follow up advice was very high. • One of the hospitals in the study area has been upgraded to medical college status, following which the faculty has increased, referrals have reduced, and infants and children who need admission were admitted to this hospital. • Families started entrusting local hospitals (study hospitals) rather than running to distant referral hospitals.
Uttar Pradesh, India	• 14 (out of 19) caregivers revealed their apprehensions that private practitioners and private hospitals extorted a large amount of money by unnecessarily admitting the children. This perception was only about formal private hospitals. • All (3/3) HCPs at secondary level facilities reported no mechanism to display bed availability in neonatal care units and paediatric wards. Moreover, references from an influential source helped get a bed in neonatal care units. This made it more challenging to access inpatient care. • OPD care involved long waiting times, unhygienic conditions, overcrowding, and unavailability of medicines at the hospital was shared by 12 (out of 16) caregivers. • All (6/6) health staff said it was stressful to manage inpatient wards, OPDs, and night shift duties with limited staff, even during normal circumstances.All (6/6) health staff said it was stressful to manage inpatient wards, OPDs, and night shift duties with limited staff, even during normal circumstances. • All (10/10) urban CHWs reported that they were new and not trained in postnatal home visits. They lacked motivation due to fewer incentives compared to rural CHWs, which affected the quality and frequency of home visits. • Also, according to 11 (out of 25) caregivers, there was no system of follow up after hospital discharge. Caregivers often stop medicines as soon as they perceive a benefit and may not adhere to the complete course.	• Despite having paediatricians in three community health centers surveyed, no paediatric services were provided during the lockdown. • Uncertainty of bed availability at higher centres increased. • Medical stores opened for limited hours. • Several private hospitals and other local HCPs increased their fees to capitalise on the opportunity due to COVID-19. • Loss of jobs along with increased medical fees by private HCPs – both trained and untrained, led to indebtedness. The patient's attendants could not find accommodation and food.	• During the stringent lockdown, regular preventive health care services such as antenatal care, identification of high-risk pregnancies, testing, etc., were halted. • 4 (out of 6) HCPs in facilities said that lack of human resources was a persistent problem in public health facilities. Additionally, the new COVID-19 ward set up increased staff reluctance to be on duty. • HCPs in 4 (out of 6) public health facilities shared that the entire focus was shifted to COVID-19 hospitals for COVID-19 patients, causing irregular supplies of antibiotics, medicines, and other essential equipment in non-COVID-19 hospitals. • Regarding OPD care, 2 (out of 6) HCPs reported that due to COVID-19, the human resource crisis had aggravated, and there was the sudden demise of many health providers who were on COVID-19 duty. • 5 (out of 6) HCPs said that the duration of OPD hours was reduced. • 7 (out of 12) staff nurses shared that they feared being quarantined and were apprehensive of the stigma associated with being diagnosed as COVID-19 positive. This fear restricted them from getting their test done for COVID-19, despite having symptoms such as cough or cold. • 1 (out of 12) staff nurse faced unfair treatment, social isolation, and rude behaviour from relatives during get-togethers such as family celebrations and ceremonies, and they were considered carriers as they were exposed to COVID-19 cases while working in a hospital on COVID-19 duties. • 17 (out of 19) mothers said that loss of job had severe implications on compliance with treatment. • 16 (out of 19) mothers shared that transportation constraints in reaching public health facilities aggravated as fares increased after lockdown. • All (25/25) CHWs were asked to do contactless visits; they were instructed not to touch babies. Therefore, they did not enter homes or touch the doors even. They would only ask the parents about the infants’ well-being from a safe distance. Parents did not take out the infants and show them to the CHWs.	• Follow-up was done on the telephone by hospital staff. • Local village doctors are available over the phone or paid home visits. • Tele-OPD mechanism by nearest/ local providers, giving medicines at doorstep, prescription over WhatsApp were initiated. • Improvement in infection control and hygiene practices was reported. • Private HCPs were generally responsive to patient needs; for example, even if the patient could not afford tests, they still attempted treatment.
Nigeria	• All primary health centers HCPs and 1 (out of 3) secondary health facility HCP reported poor condition and services of health facilities, lack of bed spaces leading to overcrowding. • Lack of separate paediatric wards or newborn units further aggravated overcrowding. • All HCPs at the secondary health facilities (3/3) reported a lack of diagnostic equipment and provision for investigations, discouraging caregivers from admitting young infants. • All facility in-charge HCPs, except one tertiary health facility, said that lack of treatment guidelines for young infants with infection had been a major factor in discouraging health workers from admitting such cases to hospitals. 2 (out of 8) facilities in-charge of the secondary health facilities and 1 (out of 3) program manager were aware of the national guideline on managing young infants with PSBI at the national level, but this was not yet adapted or institutionalised by the Kaduna State government. • The absence of paediatric specialists in secondary health facilities was a major hindrance to inpatient services. • The facility in-charge reported the chronic shortage of staff at the secondary level. All HCPs (3/3) at the public secondary health facilities said that insufficient doctors in the facilities were due to a lack of adequate staff and constant transfer (reallocation) of available ones. • All primary health center HCPs perceived and reported that treating young infants was beyond their job description, and they felt incompetent to handle sick young babies. Caregivers reported that in the OPDs, the health workers are casual and neglect the patients. • Many women were uncomfortable being attended to by male HCPs, discouraging them from accessing care for their infants. • All HCPs at primary health centers said lack of medicine supplies was a major challenge for all primary health centres regarding OPD treatment. • Complying with treatment and following referral was challenging for mothers from low economic backgrounds. • The nurse in-charge at 1 (out of 3) public secondary health facilities said that the absence of e-health technology acts as a barrier to compliance with treatment because of the long distance of tertiary hospitals from their homes.	• 3 (out of 9) facilities in-charge reported that the shortage of staff was aggravated during the pandemic due to the reassignment of staff to COVID-19 centres to manage COVID-19 patients. • Nurses and doctors at all the (3/3) public secondary health facilities said that medicines and other supplies were almost nil during COVID-19. • The high cost and non-availability of free PPE affected the HCPs. Even though the hospital authorities supplied these, the supply dwindled with the upsurge in the COVID-19 cases. Thus, HCPs reused disposable face masks or used N-95 longer than usual.	• Sudden job loss for many families led to non-compliance with treatment; 2 (out of 15) caregivers reported a loss of employment due to COVID-19, and one of them narrated how the family starved due to financial crisis. • All caregivers said that the reduced number of commercial transport options led to an increase in the cost of the few that were available. Thus, the caregivers could not afford the high travel costs. • 1 (out of 9) CHW reported a shift in their roles and responsibilities for COVID-19 duties during the pandemic, which affected their postanal home visits.	• The Kaduna State Contributory Health Management Authority policy was a functional drug supply chain for all health facilities launched by the state government. • PPE supplies were prompt and adequate in quantity. • Budgetary allocation to health care services was adjudged upwards, funds increased, and smooth fund processing without bureaucratic bottlenecks. • As part of PSBI, newborn units were created out of the pediatric wards at the two general hospitals within the study communities. • During the pandemic, the development partner donated a bus for patient care.
Pakistan	• 25 (out of 28) caregivers mentioned a lack of beds and space at paediatric wards. • 16 (out of 28) caregivers reported a lack of quality of care in the health facilities. • 3 (out of 7) policymakers and 5 (out of 14) HCPs mentioned the shortage of equipment and supplies such as incubators, ventilators, antibiotics, and investigative services at the hospitals for treating sick young infants. • Quality of care was poor in the OPD and had long waiting times. • Complying with treatment and following referrals was challenging for mothers from low-economic backgrounds.	• 2 (out of 7) policymakers said that the shortage of beds aggravated during COVID-19 as the beds were shifted to the COVID-19 ward. • 21 (out of 28) caregivers reported infants were admitted to the emergency ward instead of the paediatric ward, and families did not prefer admission to the emergency. • 3 (out of 28) caregivers said that the low quality of OPD care aggravated further. Doctors checked infants from a distance. The shift of staff during the pandemic affected the doctor-patient ratio. Waiting time was further increased.	• 14 (out of 28) caregivers reported that the fear of COVID-19 impacted their decision to seek care from health facilities, and they went for home remedies and sought care from faith healers. • 4 (out of 6) CHWs mentioned that caregivers would self-treat by using home remedies or buying medicines from pharmacies during COVID-19. • As identified by 15 (out of 28) caregivers, several new financial issues emerged regarding compliance with treatment. A spike in the doctor’s fees and the prices of medicines reduced compliance with referral advice or treatment. • 5 (out of 6) CHWs said that the caregivers prevented the CHWs from entering their homes for postnatal home visits and wanted doctors to visit home instead due to fear that the CHWs were potential carriers of COVID-19.	• In most cases, admission was not resisted; caregivers understood the importance and would consent to receive inpatient care. • Few doctors reduced their fees later and paid for medicines sometimes. • System in place for the usual procedure for treating infants; observe their condition for a few hours, check their vitals, provide medication or injections and send the baby home. • Hospitals had adequate funding to run smoothly and maintain a steady supply of medication. • Effective referral to tertiary care facility was done for infants who had severe illnesses. • A few hospitals provided transport to the tertiary care hospital and coordinated with the hospital staff to ensure that the patients received appropriate treatment. • Follow up care was also maintained at public hospitals.

ANMs – auxillary nurse-midwives, CHW – community health worker, HCP – health care provider, IPD – inpatient department, KI – key informants, OPD – outpatient department, PSBI – possible serious bacterial infection

Three-fourths of the caregivers at all sites reported poor care at the facility and limited-time interaction with HCPs, who did not touch or examine the infants. The facility staff was in a hurry to refer or discharge newborns in most sites except Ethiopia. However, an Ethiopian HCP said that the parents only brought a seriously ill infant who needed a referral to a referring facility (**Box 1** – Example 6).

Mothers reported high treatment costs due to the pandemic aggravated by unemployment, worsening their economic condition. The problem was less pronounced in Nigeria because the study population consisted predominantly of rural communities with farmers or petty traders. Small space, overcrowding, and long waiting hours were barriers that existed even before the pandemic (**Table 3**) (**Box 1** – Example 7).

Several hospitals were closed completely, offered restricted services, or were partially or completely converted to COVID-19 care centres (**Table 3**). Almost all caregivers at all sites expressed apprehension about visiting hospitals, fearing exposure and undergoing COVID-19 testing could lead to extended quarantine and stigma if tested positive (**Box 1** – Example 8).

Almost all HCPs in India and Nigeria reported the absence of clear guidelines on essential newborn care, particularly for low birth weight or preterm newborns and breastfeeding during the pandemic. There was a lack of clarity about the management of sick young infants and their testing for COVID-19. (**Box 1** – Example 9).

HCPs said they discouraged the admission of infants except in emergency. About half of the facility staff across the sites and a majority in Nigeria emphasised that district and sub-district hospitals (secondary level) functioned sub-optimally even in the pre-COVID-19 due to limited numbers of ventilators, incubators, stockouts of essential medicines and equipment, poor infrastructure, insufficient space, and inadequate laboratory facilities. Caregivers said that the waiting time was always very long, even in the pre-COVID-19 period (**Table 3**). The facility staff mentioned that the COVID-19 related supplies such as personal protection equipment (PPE), hand sanitisers, and face masks were also in short supply. At some sites, the lockdown aggravated the shortage of commodities. In UP, India, the program managers reported an acute shortage of medicines and essential supplies in non-COVID-19 designated hospitals (**Box 1** – Example 10) while, medicines for non-COVID-19 illnesses in the COVID-19 designated hospitals were being wasted, and prolonged disuse of equipment (such as incubators, radiant warmers) for routine care made some almost non-functional.

Almost all program managers saw staff shortage as a considerable challenge, which existed during the pre-COVID-19 period, except in Ethiopia, where only one-fifth cited it being a problem. Specialists/paediatricians were not available, and there was high staff turnover. Referral linkages between primary and referral facilities were lacking as only a few referral facilities were accessible. Three-quarters of HCPs in Nigeria and about a quarter each in HP and UP in India reported non-availability of treatment guidelines and clearly defined referral criteria for sick young infants even before the pandemic. Infants born within same facilities were preferred for admission in neonatal units, if needed, over those who were referred from outside for inpatient care. Some HCPs at the secondary and primary levels felt incompetent to manage sick newborns (**Table 3**).

**Figure 1:**
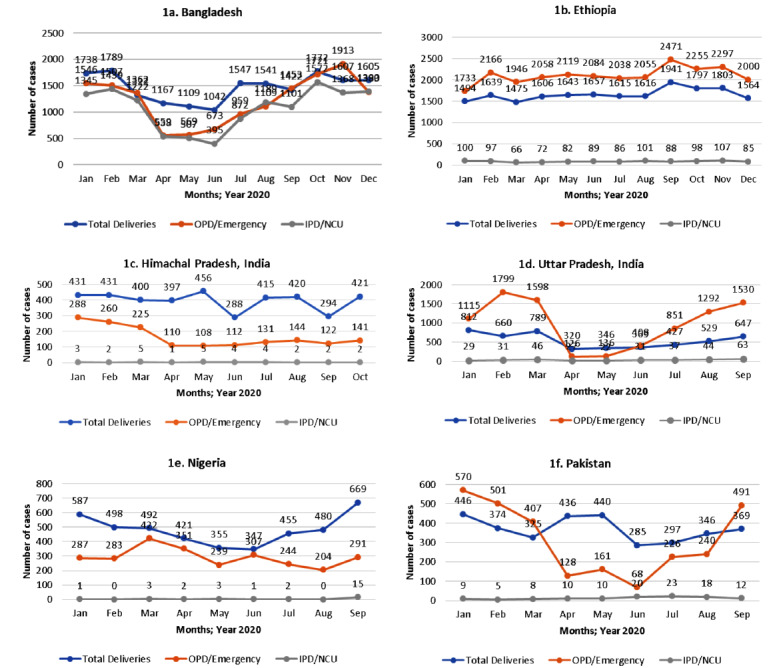
Number of deliveries, outpatient (OPD)/emergency visits and inpatient (IPD)/neonatal care unit (NCU) admissions by month at each study site.

Facility records review showed low attendance of sick young infants in the OPD/emergencies during lockdowns, but it gradually increased as lockdowns eased (**Figure 1**, panels a-f). In Pakistan, OPDs in government and some non-government facilities were closed during the lockdown. In Ethiopia, the IDIs and FGDs reflected that care-seeking for sick young infants from health facilities and CHWs was substantially affected during the lockdown. However, quantitative data did not show a considerable difference, probably due to limitations in the extracted health system data. Although strict lockdown was enforced briefly in Ethiopia, caregivers still visited the hospitals with their sick infants (**Box 1** – Example 11). Admission of sick young infants in the hospitals across all sites remained the same throughout the survey period, except in Bangladesh, where inpatient admissions were reduced during the lockdown.

### Deliveries in the secondary and tertiary care facilities

The program managers reported that the secondary and tertiary care facilities within the catchment areas handled delivery cases well during the pandemic, even those with complications. They concluded that these facilities could provide adequate intrapartum and newborn care, and that further strengthening would be helpful in improving service provision. Facility staff at some sites remarked that mothers’ agreement to their infants’ admission even during the pandemic showed the community had faith in the public health system.

The number of deliveries in the study facilities was the lowest during the lockdown at all sites compared to the pre-and post-lockdown periods (**Figure 1**, panels a-f). In HP (India), referral to higher facilities outside the study catchment areas for complicated cases was the lowest during the lockdown period, and these were managed in the local hospitals. As a result, complicated delivery rates were higher in these hospitals during the lockdown (34%) and post-lockdown (33%) period compared to the pre-lockdown period (28%) (data not shown).

A rebound in births was observed at all sites post lockdown (**Figure 1**, panels a-f). The facilities at the tertiary and a few at the secondary level appeared to be more resistant with higher resilience under the adverse circumstances during the lockdown period. Some of these facilities recruited doctors and nurses on a contract basis. The primary and most of the secondary levels facilities succumbed to the crisis. It could be due to an inadequate number of facility staff because many of them were infected with the COVID-19 infection and were in quarantine, poor infrastructure, and non-availability of equipment at the primary and secondary levels. Many primary-level facility staff were deployed for COVID duties, affecting staff availability, which was already a challenge at these facilities. Due to infrastructure issues and limited space, a separate COVID-19 unit could not be set up at the primary and secondary levels.

The negatively affected services included OPDs, normal deliveries, and inpatient treatment, including the paediatric wards, which were shut down. Only emergency services were operational. However, the footfall of patients had drastically reduced. Caregivers who used to visit government hospitals switched over to local clinics or private hospitals because of the paucity of hospital staff and fear of contracting the COVID-19 infection. Additionally, patients faced difficulties reaching the hospitals due to travel restrictions during the lockdown, affecting public transportation services.

### Effect on routine services

About four-fifths of all facility staff and CHWs across all sites and slightly less than one-fifth in Ethiopia reported that routine services such as vaccination and antenatal and postpartum care were adversely affected, and community clinics were closed during the lockdown. Caregivers were afraid of taking their children for vaccination. Outpatient care was adversely affected at different sites, either due to suspension of services or availability for limited hours, or non-availability of HCP. CHWs across all sites were not keen to carry out their responsibilities due to inadequate PPE supplies and incentives for COVID-19 duties. In Ethiopia, CHWs continued to implement their routine activities, received training on COVID-19, and engaged in COVID-19 screening and awareness-raising campaigns.

Home-based postnatal visits for newborns and referral of sick young infants were also reduced. The quality was affected due to social distancing as CHWs reported assessing newborns from a distance. All routine services in the community were stopped for 20 days during the lockdown in Bangladesh. Scheduled visits were done for 55%-75% young infants in January-March 2020, compared to 15%-53% in the lockdown and 45%-51% in the post-lockdown periods in Pakistan, UP and HP (India) (data not shown).

### Changes in hospital policies

Safety measures, such as screening and isolation of women until COVID-19 test results were available, a separate operation theatre and labour room for COVID-19 positive patients, triaging and screening of children with fever or cough, and a separate ward for COVID-19 positive children were adopted during the pandemic at most study facilities across all sites. These measures, however, often delayed treatment initiation. Keeping newborns with their mothers to promote exclusive breastfeeding was practised at some sites irrespective of COVID-19. Program managers at a few sites reported that a COVID-19 hazard allowance was introduced as an incentive for HCPs. Furthermore, referral linkages across different levels of health facilities and CHWs were strengthened by establishing effective post-discharge follow-up care, strict enforcement of hygiene and infection control practices, and the utility of mobile phones for better communication (**Box 1** – Example 12). The program managers said that round-the-clock emergency services in facilities were ensured. Most caregivers expressed complete faith in the government health system and adhered to the recommendations.

### Mitigation strategies

#### Already implemented strategies

In Ethiopia, HCPs received monetary incentives and tax relief. Hospitals providing neonatal care services were spared from changing to COVID-19 centres. Increased duty hours and a restriction on annual leave for HCPs were imposed. Transportation services/allowance were allocated for staff. In UP (India), contactless home-based postnatal visits were being conducted during COVID-19. Policy changes were implemented in HP (India) such as introducing COVID-19 testing, isolation wards, and separate operation theatres and labour rooms for COVID-19 positive women. Similarly, COVID-19 testing was mandatory for all infants with signs and symptoms of pneumonia and the establishment of special COVID-19 wards for COVID-19 positive cases. In UP and HP, India, infants were discharged earlier if they were stable than the routine period to ensure availability of space and reduce hospital exposure. They were contacted for follow-up. Many safety and precautionary measures were taken for HCPs to ensure their continued services (**Table 4 ** and **Table 5**).

**Table 4:** Common mitigation strategies across all study sites by the level of implementation at policy level, health systems level, and community and caregiver level

Policy level
**Infrastructure**
***New ideas for mitigation strategies***
• Separate infrastructure for COVID-19 and non-COVID-19 facilities. Facilities providing COVID-19 care with no scope of separate infrastructure should at least ensure that the entrance is different or the floor is different.
• 24-h hospital services to be made available for emergency care. Improve facility infrastructure and build wards for newborns.
• Creation of COVID-19 isolation centres distributed across the study area.
• Secondary and primary health centres to be upgraded and better equipped with the provision of essential amenities.
**Human resources and training**
***New ideas for mitigation strategies***
• Additional human resources would be deployed.
• Separate staff for COVID-19 and non-COVID-19 services.
• The doctor-patient ratio should be reduced.
• Regular and periodic training of staff; government is devising several approaches to enhance the capacity of staff to improve recognition of the symptoms in young infants.
• Increment in allowances, separate allowance for COVID-19 duty to all frontline workers for motivation.
• Build focus of CHWs to capture infants from 28 d to 59 d, especially after 40 d of birth, for possible serious bacterial infection (PSBI).
• Knowledge of treatment and dosing should also be conveyed to community health workers (CHWs).
**Service availability**
***New ideas for mitigation strategies***
• Need appropriate guidelines for the care of sick young infants, routinely and during COVID-19.
• Support telemedicine services for treatment and follow-up.
• Supplies of essential medicines.
***Mitigation strategies in the pipeline***
• Presence of investigative services and the availability of oxygen.
• Availability of supplies of essential medicines at facilities.
• Availability of adequate quantity of personal protective equipment (PPE) for staff, patients, and attendants at facilities.
• Increase frequency of COVID-19 testing of hospital staff to rule out infection.
**Medicines and supplies**
***New ideas for mitigation strategies***
• Increase the supply of medicines across all facilities in inpatient wards, emergencies, neonatal care units and outpatient departments (OPDs).
**Transportation**
***New ideas for mitigation strategies***
• Free and adequate ambulances should be made available round the clock.
• Appropriately sanitised to increase utilisation.
**Financial**
***New ideas for mitigation strategies***
• There should be subsidised treatment available for the poor.
• Increased availability and frequency of funds for poor patients to access private services.
• Decrease bureaucratic challenges associated with accessing welfare funds for poor patients.
• Linkages of caregivers to compensatory schemes for treatment relief packages; linking caregivers with incentive-based schemes.
**Awareness creation**
***New ideas for mitigation strategies***
• Information, education, and communication activities need to be promoted to create awareness and spread the information on danger signs or PSBI signs requiring immediate care-seeking from appropriate sources.
• Policymakers highlighted the importance of collaboration among various ministries to work together and a multisectoral approach to raise public awareness about childhood illness at the community level, such as engaging religious leaders to announce from the local religious place (such as Mosque) loudspeaker.
**Collaboration with private facilities and their orientation**
***New ideas for mitigation strategies***
• A strong public-private-partnership would be built by outsourcing private doctors who are willing to provide services in the OPD of government hospitals.
• Support through research projects would help ensure appropriate management of PSBI in young infants. The research team may also facilitate COVID-19 vaccination for frontline health workers.
**Health systems level**
**Infrastructure**
***New ideas for mitigation strategies***
• Waiting space should be expanded; widening space between beds and delivery couches to minimise the risk of COVID-19 infection.
***Mitigation strategies are already being implemented***
• Appropriate hygiene and sanitation to be maintained.
***Mitigation strategies in the pipeline***
• Separate floor was allocated for COVID-19 treatment so regular patients would not be affected.
**Human resources**
***New ideas for mitigation strategies***
• Negotiations with the administration to ensure the availability of staff.
• Adequate training and skill development of all staff.
**Transportation**
***New ideas for mitigation strategies***
• Separate ambulance for transportation of third-trimester pregnant women for antenatal care /sick young infants with PSBI and separate for COVID-19 confirmed cases with social distancing.
**Services**
***New ideas for mitigation strategies***
• Hospitals should not be closed; routine care should not be disrupted.
• Strengthening inter-facility communication and coordination for referrals.
• Establish an appropriate referral chain from home to tertiary care with follow up of referred patients.
• Extend doctors and staff service hours at primary and secondary care facilities and ensure a full-time qualified doctor at the primary health facilities. All remunerations and incentives to be paid on time for all health staff.
**Functioning of hospitals and protocols**
***New ideas for mitigation strategies***
• Before providing treatment, standard operating procedures (SOPs) should be followed in hospitals.
• Triage should be established for infants with danger signs.
• Doctors and nurses provide counselling on taking the course of medicine. If doctors are unable to provide a complete treatment course, then recommend caregivers to buy outside the facility.
***Mitigation strategies in the pipeline***
• Limiting visitation of attendants/relatives. All patients may be advised not to come for routine visits to the OPD if it can be avoided or postponed during COVID-19 to prevent exposure.
• Pharmacy counters may be increased.
• Strict restrictions followed. lesser number of patients, severe cases attended to, antenatal care services restricted.
***Mitigation strategies are already being implemented***
• Infants were discharged earlier, if they were stable to ensure availability of space, contacted and called for follow up.
• Many safety and precautionary measures are taken for staff in facilities to ensure that they continue to come to the health facilities.
• Rosters have been introduced to limit the number of staff members.
**Use of mHealth and digital technology**
***New ideas for mitigation strategies***
• Digital platform for standardised assessment and referral of young infants; to train facility staff.
• Adequate training of hospital staff and CHWs to be provided with appropriate equipment such as a handheld pulse oximeter that measures pulse, oxygen saturation and counts respiratory rate. If oxygen saturation falls, the infant can be immediately referred to the health facility.
• Telemedicine, E-counselling and video conferencing were suggested for treatment and follow up.
**Effective supervision**
***New ideas for mitigation strategies***
• A robust supervisory committee constituted with community ownership and telephonic supervision of CHWs by health providers in facilities was suggested.
**Quality of services**
***New ideas for mitigation strategies***
• Hospital staff should be careful in behaving with patients and caregivers appropriately. Most complaints are against lower-level staff.
• Suggestion to monitor staff activities through surprise visits and talking with clients.
• Regular monthly meeting to discuss any complaints about any staff and motivate doctors, nurses and other staff was suggested about their work.
**Community and caregiver level**
**Use of mHealth and digital technology**
***New ideas for mitigation strategies***
• Digital technology to train CHWs. The CHWs teach about danger signs to families to recognise danger signs.
• CHWs followed up with families over the phone when they could not visit the home.
• Tele-consultation facility for CHWs and family on young infant care backed with a digital danger-sign assessment tool and guidance on referral assistance and follow up care.
• CHWs' dependency on caregivers’ reporting will be addressed by backing them up with a digital application, improving confidence in assessment and facilitating referral.
**Training of CHWs by their supervisors**
***New ideas for mitigation strategies***
• Strengthen CHW skills for newborn danger signs assessment and home-based postnatal visits training of urban CHWs and their orientation.
• PPEs for all CHWs should be ensured and supportive environment for CHWs during the early stages of identification to care-seeking for the referral provided. Standardisation exercises should be conducted to enhance clinical skills in identifying danger signs.
• Initial and periodic training (virtual during COVID-19) and handholding of CHWs and supervisors on managing sick young infants during health emergencies such as COVID-19, CHWs will be equipped with information to do their home-based postanal visits and infant care without compromising quality. These sessions will generate new knowledge on the felt need of CHWs for such health emergencies.
**Community awareness**
***New ideas for mitigation strategies***
• IEC activities to promote awareness, impart correct knowledge through simple messaging to reduce panic and anxiety around COVID-19 and address fear and rumours around hospital safety and COVID-19 tests. Existing government community platforms at each site would be used to conduct these activities.

ANC – antenatal care, ANMs – auxillary nurse-midwives, CHW – community health worker, HCP – health care provider, HR – human resource, IEC – information, education, and communication, IPD – inpatient department, NICU – neonatal intensive care unit, OPD – outpatient department, PDSA – plan-do-study-act, PHCs – primary health centres, PSBI – possible serious bacterial infection, SHG – self health group, SOPs – standard operating procedures

**Table 5:** Site-specific strategies by the level of implementation at policy level, health systems level, and community and caregiver level

Policy level
**Bangladesh**
***Mitigation strategies in the pipeline***
• The doctors at the public hospital have discussed the existence of a social welfare fund used to help needy patients to buy medicines or transfer to another hospital.
**Ethiopia**
***Mitigation strategies are already being implemented***
• Monetary incentives were given to staff, tax relief.
**Himachal Pradesh, India**
***Mitigation strategies are already being implemented***
• Women who visited hospitals were tested for COVID-19 and kept in the isolation ward till the results were declared. If negative, they were shifted to the regular ward. If positive, they were kept in the isolation ward.
• A separate operation theatre and labour room designated for positive women.
• For babies, no separate isolation ward, babies were kept with their mothers wherever they were placed, and breastfeeding was promoted even if mothers were positive.
***New ideas for mitigation strategies***
• Greater access to Kilkari and LokMitra (the Web-enabled Government- Citizen Interface) videos and use of audio-visual aids such as television and telemedicine.
• Updating the ‘Mother and Child Protection’card with information on danger signs and pictorial representation for non-literate mothers.
• Plan-Do-Study-Act (PDSA) cycle; program managers reported feasibility to implement the study, no problems foreseen. There is a need to start the study, identify the gaps and resolve the barriers. Before starting, they need to make a good implementation plan, distribute responsibilities, and provide the required training.
• A mobile clinic was suggested to reach out to the community for managing sick young infants.
• Doctors working at government facilities in these remote areas should be paid higher salaries and better working conditions for sustained motivation. Permanent positions in the health facilities, once filled, should be retained for at least 3 y for increased accountability.
• Increase access to health centres by relocating buildings in non-residential regions on top of the hill, rendering them redundant and non-functional. The number of sub-centres for one PHCs should be increased in the difficult geographical terrain. • Establish Mohalla (neighbourhood) Clinics to provide primary medical care closer to home.
• Infants who present with signs of pneumonia are tested for COVID-19. If negative and if the infant needs admission, he/she is admitted to the general ward. If positive, they are admitted to special COVID-19 wards.
• Government to establish a system of a loan at minimum interest to facilitate care-seeking and treatment by poor people. The initiative can be taken by the Self Help Group (SHG).
• Mahatma Gandhi National Rural Employment Guarantee Act”, Indian labour law and social security measure that aims to guarantee the 'right to work, needs to be implemented more effectively to counter the unemployment problems. More small and micro industries should be encouraged for employment.
• Health insurance for health staff and frontline workers should be made mandatory, and village (Gramin) banks should be opened to give loans to the public.
**Uttar Pradesh, India**
***Mitigation strategies are already being implemented***
• Contactless postnatal home visits to be conducted during COVID-19.
***New ideas for mitigation strategies***
• Creation of a district-level review mechanism for infant health care for efficient referral linkages, reducing variations in treatment algorithm, addressing HR and supply issues obstructing PSBI diagnosis, treatment and management in health facilities.
• Engaging district-level health systems -leadership through the district health system for review and supporting COVID-19 aggravated infant health care issues. This platform will be actively engaged for reviewing, updating and working together on solutions for COVID-19-related barriers.
**Nigeria**
***Mitigation strategies are already being implemented***
• Establishment of directorates at all levels of health care and WhatsApp and zoom platforms to discuss the number of COVID-19 cases and situations.
• The 10-a-day COVID-19 screening/testing policy to have a clearer picture of the COVID-19 situation was established by the Ministry of Health. Through this policy, the primary health centers must submit at least 10 specimens for COVID-19 testing every day in the States.
• The state resorted to using hotels for an overwhelming load of patients.
**Pakistan**
***New ideas for mitigation strategies***
• Issues related to housing, sewage lines and pollution need to be overcome.
• Those who are unemployed need jobs.
• Relief from the government and employment opportunities should be created. Respondents who primarily worked in the fishing industry had many grievances owing to their financial conditions and needs.
**Health systems level**
**Ethiopia**
***New ideas for mitigation strategies***
• Telegram group to communicate and give a technical update to regional child health focal persons. Campaigns through broadcasting television and radio spot message developed.
***Mitigation strategies are already being implemented***
• Hospitals providing NICU services were spared from changing to COVID-19 centres. Offloading of cases, shifting patients to low caseload hospitals and health centres.
• Changing the staff time – start service early in the morning; Restriction on allowing annual leave for health workers.
• Transportation services (allowance) allocated for staff. Walking by foot to health facilities because of a shortage of public transport was encouraged and helped.
**Himachal Pradesh, India**
***New ideas for mitigation strategies***
• Nurses from local areas to be recruited would be expected to have long term commitments than those recruited from outside the state.
• Telephonic cross-check by doctors to ensure that CHWs do the home visits and educate families.
• The telemedicine portal is operational. These are linked with the faculty directly. The number 104, a dedicated helpline pan HP, is functional during working hours. Need a 24x7 helpline toll free number with a panel of doctors where the caregivers could call for seeking care; in discussion with the state.
• Alternative means of transportation, such as the doli (palanquins) system and bike ambulances, need to be strengthened. The government will provide 2 or 3 more ambulances for PSBI.
• Task shifting and public-private partnership were suggested. Axillary nurse-midwives are trained to administer injectables, and they could be trained to provide PSBI injectable treatment.
***Mitigation strategies are already being implemented***
• The information, education and communication (IEC) material has been done in other areas, promotion of zinc and ORS in diarrhoea, immunisation coverage, a lot of media coverage, and awareness creation campaigns have been conducted.
**Uttar Pradesh, India**
***New ideas for mitigation strategies***
• For Green zone districts or green zone areas of orange zone districts*, conduct on-site OPD follow up with social distancing; For Orange zone/ Red zone/ areas, conduct telephonic follow up.
• System for teleconsultation.
• 24/7 toll-free telecommand center for efficient referral, tracking of infants, follow-up care.
• Capacity strengthening of doctors and nurses at community health centers to manage young infants with PSBI and management through online/physical visits by experienced paediatricians. PSBI services offered through community health centers will build trust with the community, win the confidence of CHWs, and build skills among community health centers’ staff.
• In birthing facilities, especially those with high delivery load, ensure that all mothers are educated on danger sign recognition and where to seek care.
• Private and charitable hospitals can be engaged to follow the same protocol for assessment and referral of YI with PSBI signs – these will act as positive influencers.
• Design protocols for PSBI identification and management at each level of care in the health system (eg, CHW, Auxiliary Nurse Midwife, community health centers, district hospital, etc.).
**Nigeria**
***Mitigation strategies are already being implemented***
• The ‘255-ward’ service policy was designed to ensure that every political ward, the smallest geographical demarcation politically, has an upgraded facility that will serve as the first referral level for the primary health care facilities.
• The government embarked upon community testing to increase the number of people tested as a deliberate policy, with the hope that if a large number of people are tested negative, the fear that every sick person is likely to have COVID-19 will be reduced and consequently build confidence for both families and health worker, leading to improved utilisation of the health services.
• Follow up care was ensured through sensitisation by community workers and home visits, regular Ward Development Committee/Maintaining contact through a phone call, volunteer community mobilisers to visit families at home where possible and set up a follow-up group.
• Many other facilities created new ways of protecting their staff and patients; all new patients entering the facility would go straight to the filter station, where mandatory temperature checks were done before accessing any service.
• Establish partnership with transport workers and a non-government organization.
***Mitigation strategies in the pipeline***
• Rehabilitating the tricycle initiative to help facilitate patient referral.
***New ideas for mitigation strategies***
• Deliberate withholding patient discharges until they show evidence of purchasing their discharge drugs; to ensure that such patients have enough drugs to complete their discharge prescriptions.
**Community and caregiver level**
**Himachal Pradesh, India**
***New ideas for mitigation strategies***
• Panchayat members (community leaders) are well respected, and people listen to them, so they should be the ones to relay any information even regarding child health.
• Mothers are to be counselled at all opportunities, such as post-delivery discharge, ANC, and immunisation sessions.
• A mobile-based application can be developed with videos on danger signs, including ways to measure oxygen saturation. Families can be oriented to identify danger signs using this app. CHWs can use this to identify PSBI signs, classify infants as having sepsis or not, and document them in the database.
• CHWs should be given cycles for travelling around the village and from one village to another.
• Male health workers can be engaged during the COVID-19 pandemic and subsequent lockdown if it occurs instead of CHWs so that the CHWs can conduct their routine duties on time.
**Uttar Pradesh, India**
***New ideas for mitigation strategies***
• The formation of community advisory groups of volunteering untrained health service providers (quacks) and faith healers to encourage early care-seeking of sick young infants from skilled health facilities was proposed. They can be trained in identifying PSBI infants for referral; prior administration of antibiotics can be discouraged unless the infant is critically ill.
• Initiatives to overcome CHWs’ COVID-19 attached stigma: availability of a self-testing kit for CHWs will enable them to self-check for COVID-19 infection regularly, which they can show to their beneficiaries to gain entry. Ensure that all CHWs are vaccinated for COVID-19 protection. CHWs may also carry COVID-19 negative certificates with limited validity.
• Design and development of information materials/ IEC for awareness generation and counselling: IEC materials, eg, social media-friendly posters and leaflets, can be developed for different audiences. These materials should be available for physical distribution, posting, and sharing on social media platforms. These can be posted in birthing facilities.

ANC – antenatal care, ANMs – auxillary nurse-midwives, CHW – community health worker, HCP – health care provider, HR – human resource, IEC – information, education, and communication, IPD – inpatient department, NICU – neonatal intensive care unit, OPD – outpatient department, PDSA – plan-do-study-act, PHCs – primary health centres, PSBI – possible serious bacterial infection, SHG – self health group, SOPs – standard operating procedures

*As per the guidelines issued by the state government, districts and geographical areas within a district were declared as ‘Green Zone’, where no COVID-19 positive cases were reported, ‘Orange Zone’, where less than 15 COVID-19 positive cases were reported, and ‘Red Zone’or ‘Hot spot’, where >15 COVID-19 positive cases reported.

#### Strategies that are in the pipeline

A few program managers informed that strategies such as establishing the COVID-19 isolation centres, separate floors for COVID-19 care, and limiting attendants for admitted patients were in the pipeline. Giving incentives to HCPs to perform COVID-19 duties, conducting frequent COVID-19 tests, ensuring adequate supplies of PPE, building social welfare funds for those in need, conducting web-enabled community awareness, and rehabilitating local means of transport such as the tricycle initiative in Nigeria were also being planned as mitigation strategies.

#### Ideas for new strategies

Almost all program managers and HCPs (except in Ethiopia) said that for early identification of PSBI, information, education, and communication activities were critical for awareness of danger signs in neonates requiring immediate care-seeking from appropriate sources, utilising existing government platforms. They said that door-to-door health awareness messages through CHWs would be continued during the pandemic.

Program managers in HP, India, and Nigeria suggested alternative means of transportation to improve care-seeking by strengthening the indigenous options of ‘*doli’* system (palanquins), bike ambulances, and tricycle initiatives to transfer patients in the absence of ambulances, especially on narrow roads unsuitable for four-wheelers (**Box 1** – Example 13). High compliance with safety measures and appropriate sanitisation in the facilities and ambulances would ensure staff safety and improve service utilisation as reported by HCPs.

Program managers, facility staff, and KIs unanimously recommended the establishment of separate infrastructure and staff for COVID-19 and non-COVID-19 care. Where this was not possible, they suggested separate facility entrances and floors housing these patients as an alternative. Mobile clinic to treat sick young infants was another popular suggestion by the program managers in India. Audio and video calls, availability of round-the-clock toll-free numbers with access to a panel of doctors, developing simple digital applications and video-based tutorials on prevention and management of infections, telegram groups, and online meetings for HCPs; which would improve the management of sick young infants during the pandemic was also suggested (**Box 1** – Example 14).

Program managers also listed filling staff vacancies, adequate training, including frequent virtual training sessions for CHWs, appropriate tools, and adequate supervision of CHWs to improve their performance (**Box 1** – Example 15). They recommended standardised training for all CHWs in managing sick young infants. Additionally, they said that using digital screening tools, such as handheld pulse oximeters and a digital platform to follow sick young infants closely, would improve the performance of CHWs, which would be helpful even after the pandemic.

The constitution of a competent local supervisory committee for enforcing social accountability was also suggested. Almost all program managers reported that appropriate incentives for COVID-19 duties were urgently needed to improve staff motivation. They recognised that service quality was negatively impacted during the pandemic due to stress, anxiety, fear, fatigue, excessive workload, and exhaustion from wearing PPE for prolonged periods. Extending facility service hours, appointmenting qualified doctors at primary health care centres, increasing job tenure with less frequent transfers of senior doctors were suggested, along with increasing the number of health centres proportionate to the population and establishing neighbourhood clinics with the provision of essential services to improve long-term treatment access. In HP (India), the program managers added that strict actions were needed against people who harassed and humiliated frontline HCPs during the pandemic.

Program managers, in general, emphasised the need for urgent improvement of service quality. They recommended capacity strengthening and motivation of HCPs through online platforms or on-site visits by senior paediatricians from tertiary level hospitals. Some program managers in India suggested task shifting with the training of Auxiliary Nurse-Midwives to administer injectable antibiotics to young infants and engaging private doctors through public-private partnerships as beneficial strategies.

All program managers and HCPs considered the adequate and continuous supply of medicines and other commodities essential for inpatients and OPDs. Additionally, funding assistance from not-for-profit research institutes or local organisations would be helpful. Support for transportation of sick young infants to the hospital and the presence of a dedicated toll-free number would improve referral. Facilitating staff vaccination would reduce the burden on the government.

The need for free or subsidised treatment provision was reported across all sites by caregivers and KIs because of increased travel and treatment costs and financial stress due to loss of employment. Providing financial schemes through government-established loans (with low-interest rates) would also facilitate care-seeking and treatment by poor people. As a long-term strategy, irrespective of the pandemic, around half of the program managers suggested small and micro industries for employment, provision of health insurance to staff, ensuring fund availability for needy patients to access private services, and decreasing bureaucratic challenges associated with accessing welfare funds by the poor could be valuable strategies to improve health services and utilisation.

## DISCUSSION

This formative research provided valuable insights into the challenges of managing sick young infants during the COVID-19 pandemic. Many existed before the pandemic [13-26], but became pronounced during the pandemic. The key challenges were: lack of awareness on early identification of serious illnesses requiring care-seeking from sources outside the home, use of home remedies leading to delay in care-seeking, access barriers due to long distances and inadequate or expensive transportation, suboptimal treatment at health facilities due to poor infrastructure, inadequate human resources, unavailability of essential equipment, limited laboratory support, and insufficient medicines and other supplies. The new normal, characterised by restricted movement, reduced transportation, limited-service availability at facilities for illnesses other than COVID-19, and resource reallocation, impacted appropriate treatment for sick young infants. Additionally, fear of COVID-19 infection in the hospital, limited community activities by CHWs, and increased financial stress affected prompt care-seeking. However, it was encouraging that several mitigation strategies were proposed to manage sick young infants across all study sites.

The sub-optimal functioning of health systems in LMICs is known. This research provided an independent understanding of the functioning of the health systems at these sites and obtained profound insights into the possible reasons for barriers and challenges. Some, inherent to the health systems at the various study sites, were expected to persist post-pandemic, impeding access to appropriate care. The care-seeking and care provision were affected due to the pandemic. Essential health care services were disrupted, access was further restricted, and out-of-pocket expenditures escalated due to exorbitant rates for private transportation and increased cost of treatment in private facilities. Loss of employment precipitated financial stress for almost all families.

Limited data are available on the challenges in managing sick young infants in LMICs during the COVID-19 pandemic. A recent survey revealed stress among HCPs and indicated the absence of clarity and guidelines regarding the care of newborns during the pandemic [33]. Another explored the effect on maternity services during the COVID-19 pandemic’s initial stages, when over 700 maternity workers reported antenatal and postnatal care reductions and a shift in birth location from hospital to home [34]. They reported compromised quality of care, particularly evidence-based respectful care practices such as birth companions, family visitation, keeping newborns and mothers together, and breastfeeding. A higher workload due to staff shortages, longer shifts, and increased stress levels were reported [33]. Most data was from maternity HCPs, with less than ten respondents from the neonatal care domain.

There is concern that the negative impact of the COVID-19 pandemic and lockdown on care-seeking practices and CHWs’ performance may become a permanent community behaviour (post-COVID). Many caregivers who shifted to faith healers, alternate systems of health care, formal and informal private sector, or home remedies might consider these as better options closer to home. Another concern was getting government services back on track, such as appropriate home-based CHWs’ postnatal care visits and quality of care at the government hospitals. However, our data were reassuring, indicating that the community returned to pre-COVID-19 practices. Once the lockdowns were lifted, the number of infants visiting the facilities and institutional deliveries reverted to almost pre-COVID-19 levels.

Amidst all the reported challenges, some positive aspects were brought forth. Despite the difficulties, the community sustained faith in the government health system and complied with medical advice for inpatient or outpatient treatment at most sites. Community members at a few sites who started utilising local hospitals due to limited access to far-off tertiary care hospitals were generally satisfied, reinforcing their faith in the health systems. Additionally, even during the COVID-19 pandemic, routine services such as immunisation, antenatal care, and postnatal home visits, although reduced, did not stop entirely. During the post-lockdown period, the situation started improving but did not reach the pre-lockdown status by the end of the study, which is about three months of follow-up data collection. As reported by HCPs and program managers, an unexpected pandemic-related finding was the reduction in the burden of illnesses and seasonal vector-borne infections such as malaria and dengue, although quantified evidence was outside the scope of this study.

Mitigation strategies emerged from HCPs and beneficiaries based on real-life experiences, unlike the top-down approach of identifying solutions. Some of the recommended strategies, such as separate dedicated infrastructure, human resources, exclusive ambulance services for COVID-19 patients, COVID-19 screening of all patients reporting to the facilities, and shifting out COVID-19 positive cases could reduce the spread of infection. Other strategies such as reassuring the community of hospitals’ safety, conducting extensive community awareness programs, taking strict actions against people harassing and discriminating frontline health workers, as well as giving out incentives, health insurance, appropriate PPEs, and vaccines for HCPs would increase service utilisation and staff motivation. As suggested by the private providers, strategies for retaining good doctors in the government sector and public-private partnerships in hard-to-reach remote areas may be considered long-term solutions. In the absence of conventional ambulances, the alternative transportation options with indigenous innovations are worth exploring. Mobile apps and handheld equipment for community-level workers, telemedicine, audio-visual aids, and digital platforms for case identification, treatment, follow-up care, and supportive supervision can be tested for feasibility and may be helpful even beyond the pandemic. Community empowerment, ownership, and participatory actions through the constitution of supervisory committees with community representations, and resource mobilisation for the neediest, as suggested by respondents, could be promising for generating demand and ensuring equitable health service access and utilisation.

Our study has some limitations. The quantitative data were collected retrospectively, so recall bias in reporting cannot be overlooked. Further, the quantitative data were collected concurrently from the records in the facilities and records of the CHWs. Due to the disruptions caused by the pandemic, there is a possibility that the data may not have been captured rigorously with missing data, which could be the reason for the discrepancy between the qualitative and quantitative data. It was not possible to confirm data reliability, as it was not a prospective process. At some sites, FGDs could not be conducted because of social distancing norms. The qualitative method had to be restricted to IDIs and observations only.

The study strengths included obtaining information from multiple stakeholders for comprehensive insights. The study was conducted across six sites in five LMICs, generating rich qualitative information from various contexts. The researchers did not drive the solutions and mitigation strategies. These emerged from various stakeholders, including public and private HCPs at different levels and the end-users, who could share their thoughts. Such solutions may be useful to consider in the long run for similar settings. Many suggested strategies were beyond the pandemic challenges, as these were inherent barriers within the health systems under normal conditions.

## CONCLUSIONS

Sick young infants are among the most vulnerable groups needing protection during the pandemic. Our findings provide valuable insights for policymakers. There is an urgent need for clear guidance for the care of normal and sick young infants during the pandemic. It also indicates that the health systems need to be flexible and adaptive. Decentralised decision-making autonomy, ability to mobilise funds or use untied funds with appropriate documentation, procurement of supplies as and when needed, simple operational strategies to recruit and train staff at short notice, and many other pragmatic solutions to optimise operationalisation emerged. Our research provides important insights into the preparedness of health systems facing such catastrophes. While some of the solutions are aspirational and long-term, some are feasible and implementable immediately without substantial investments. The long-term strategies may have implications for crucial policy level changes to strengthen the health system and effectively deal with such public health challenges in the future.

## Additional material


Online Supplementary Document
