# Supplementary material for: Barriers to optimal care and strategies to promote safe and optimal management of sick young infants during the COVID-19 pandemic: A multi-country formative research study
Source: J Glob Health. 2022 Sep 3;12:05023. doi: 10.7189/jogh.12.05023 (PMC9440476; doi:10.7189/jogh.12.05023)
Supplement: Online Supplementary Document [file jogh-12-05023-s001.pdf]

Supplementary Figure S1. Conceptual framework of possible factors influencing PSBI management due to the COVID-19 pandemic

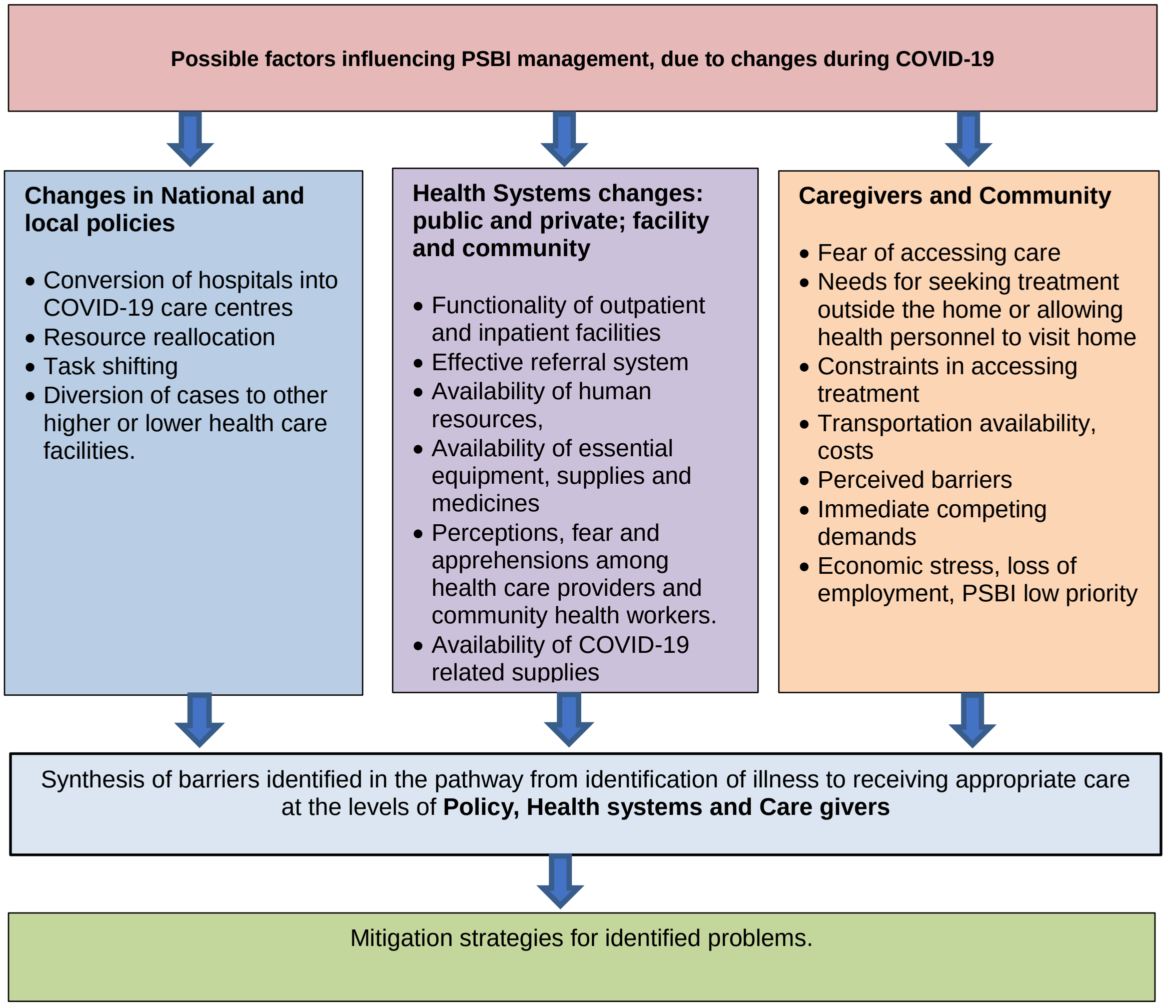

Supplementary Table S1. Site description

**Site**	**Bangladesh**	**Ethiopia**	**UP, India**	**HP, India**	**Nigeria**	**Pakistan**
Study area in square kilometers	3452	625*	1100	2825	2629	2590
Population	~ 3,434,188	~ 1,270,472	~ 1,900,000	~ 579,942	~ 550,000	~ 4,980,527
Population type	Urban, rural	Urban, rural	Urban and rural	Peri-Urban	Peri-urban, Rural	Urban, rural
Number of study facilities	4	2	3	2	3	2
Level of study facilities	Two secondary level hospitals and two tertiary level hospitals	Two secondary level hospitals	One tertiary level and two secondary-level hospitals	One tertiary level and one secondary-level hospital	Three secondary level hospitals	One tertiary level and one secondary-level hospital
Number of total beds in study facilities	Secondary level hospitals: 50 l<br>Tertiary level hospitals: 500-1000	Secondary level hospital: 301<br>Tertiary hospital: 346	Secondary level hospitals: 50 – 100 beds/hospital<br>Tertiary level hospital: 1000	Tertiary level hospital: 250<br>Secondary level hospital: 100	Giwa General Hospital: 175<br>Hajiya Gambo Sawaba General Hospital: 200<br>Major Ibrahim Bello Abdullahi General Hospital: 75	Secondary level hospital: 122<br>Tertiary level hospital: 81
Number of paediatric beds in study facilities	Secondary level hospitals: not specified<br>Tertiary level hospital: 80	Secondary level hospitals: 83<br>Tertiary level hospitals: 116 beds	Secondary level hospitals: 19<br>Tertiary level hospital: 120	Tertiary level hospital: 35<br>Secondary level hospital: 21	Giwa General Hospital: 80<br>Hajiya Gambo Sawaba General Hospital: 25<br>Major Ibrahim Bello Abdullahi General Hospital: 0	Secondary level hospital: 110<br>Tertiary level hospital: 0
Neonatal intensive care units in study facilities	Secondary level hospitals: not available<br>Tertiary level hospital: available	Available in all hospitals	Available in all hospitals	Available in all hospitals	Not available at all hospitals	Secondary level hospital: not available<br>Tertiary level hospital: available

***** Only main towns were included to estimate the study area, rural catchment areas were not included
